# Gas Sensors Based on One Dimensional Nanostructured Metal-Oxides: A Review

**DOI:** 10.3390/s120607207

**Published:** 2012-05-30

**Authors:** M. M. Arafat, B. Dinan, Sheikh A. Akbar, A. S. M. A. Haseeb

**Affiliations:** 1 Department of Mechanical Engineering, Faculty of Engineering, University of Malaya, 50603 Kuala Lumpur, Malaysia; E-Mail: arafat_mahmood@siswa.um.edu.my; 2 Department of Materials Science and Engineering, The Ohio State University, 2041 College Road, Columbus, OH 43210, USA; E-Mails: dinan@matsceng.ohio-state.edu (B.D.); akbar.1@osu.edu (S.A.A.)

**Keywords:** gas sensor, one dimensional nanostructures, metal-oxides

## Abstract

Recently one dimensional (1-D) nanostructured metal-oxides have attracted much attention because of their potential applications in gas sensors. 1-D nanostructured metal-oxides provide high surface to volume ratio, while maintaining good chemical and thermal stabilities with minimal power consumption and low weight. In recent years, various processing routes have been developed for the synthesis of 1-D nanostructured metal-oxides such as hydrothermal, ultrasonic irradiation, electrospinning, anodization, sol-gel, molten-salt, carbothermal reduction, solid-state chemical reaction, thermal evaporation, vapor-phase transport, aerosol, RF sputtering, molecular beam epitaxy, chemical vapor deposition, gas-phase assisted nanocarving, UV lithography and dry plasma etching. A variety of sensor fabrication processing routes have also been developed. Depending on the materials, morphology and fabrication process the performance of the sensor towards a specific gas shows a varying degree of success. This article reviews and evaluates the performance of 1-D nanostructured metal-oxide gas sensors based on ZnO, SnO_2_, TiO_2_, In_2_O_3_, WO_x_, AgVO_3_, CdO, MoO_3_, CuO, TeO_2_ and Fe_2_O_3_. Advantages and disadvantages of each sensor are summarized, along with the associated sensing mechanism. Finally, the article concludes with some future directions of research.

## 1. Introduction

Semiconducting metal-oxides are promising candidates for gas sensing applications because of their high sensitivity towards many target gases in conjunction with easy fabrication methods, low cost and high compatibility with other parts and processes [1–4]. To date, ZnO, SnO_2_, TiO_2_, In_2_O_3_, WO_3_, TeO_2_, CuO, CdO, Fe_2_O_3_ and MoO_3_ nanostructures have been developed with different dimensions and sensor configurations. It was found that both the surface state and morphology of the metal-oxides play important roles in gas sensing performance [5]. Depending on the application of interest and availability of fabrication methods, different surface morphology and configurations of the metal-oxides have been achieved; including single crystals, thin films, thick films and one dimensional (1-D) nanostructures [6]. Of these, 1-D nanostructures have attracted much attention in recent years because of their potential applications in gas sensors [7]. 1-D nanostructures are particularly suited to this application because of their high surface-to-volume ratio as well as their good chemical and thermal stabilities under different operating conditions [8,9].

Development of fabrication methods for producing 1-D nanostructures has been a major focus in the field of nanoscience and nanotechnology [10]. Several routes have been investigated for 1-D metal-oxide nanostructures for gas sensing applications. These include hydrothermal [11], ultrasonic irradiation [12], electrospinning [13], anodization [14], sol-gel [15], molten-salt [16], carbothermal reduction [17], solid-state chemical reaction [18], thermal evaporation [19], vapor-phase transport [20], aerosol [21], RF sputtering [22], molecular beam epitaxy [23], chemical vapor deposition [24], nanocarving [25], UV lithography and dry plasma etching [26]. Depending on the processing route and treatments, different types of nanostructures with different surface morphology can be achieved. Some examples of nanostructures produced by these methods include nanorods [5,7], nanotubes [14], nanowires [17], nanofibers [13], nanobelts [22], nanoribbons [27], nanowhiskers [28], nanoneedles [29], nanopushpins [30], fibre-mats [21], urchins [31], and lamellar [32] and hierarchical dendrites [20]. However, these variations in morphology showed a varying degree of success at detecting different types of reducing and oxidizing gases such as H_2_, H_2_S, NH_3_, CO, NO_2_, O_2_, liquefied petroleum gas (LPG), ethanol, methanol, xylene, propane, toluene, acetone and triethylamine.

The sensor's response to a given gas can be enhanced by the modification of both surface states and bulk properties of the 1-D metal-oxide nanostructures. These modifications can be achieved by either depositing nanoparticles on the nanostructure's surface, or coating and doping with impurities. Sensors utilizing these types of surface and bulk property modifications showed somewhat higher sensitivity compared to unmodified systems.

This article presents a comprehensive review of the recent research efforts, developments and approaches for the fabrication of 1-D metal-oxide gas sensors. The fabrication of gas sensors with 1-D nanostructures is described along with a discussion of sensing performances. The current model and theories describing the gas sensing mechanism is also introduced for 1-D metal-oxide nanostructures. Finally, key findings are summarized and possible future developments in 1-D metal-oxide gas sensors are presented.

## 2. Gas Sensor Performance Characteristics

Semiconducting materials generally owe their conductivity to their deviation from stoichiometry [33]. Interstitial cation and anion vacancies also play an important role in the conductivity [33]. In general, semiconductor metal-oxide sensors operate by virtue of gas adsorption on the surface that leads to a change in the electrical resistance of the oxide. Based on the charge carrier, semiconducting materials can be divided into two groups: n-type (electrons are major carrier, such as ZnO, SnO_2_, TiO_2_, In_2_O_3_, WO_x_, AgVO_3_, CdO and MoO_3_) and p-type (holes are major carrier, such as CuO, NiO and TeO_2_) materials. Target gas species can also be classified into two groups: oxidizing gas or electron acceptors such as O_2_, NO_2_ and reducing gas or electron donor such as H_2_, H_2_S, HCHO, CO and ethanol. When a reducing gas is chemisorbed on the surface of an n-type material, extra electrons are provided to the material surface. As a result the resistivity of n-type material is decreased. The opposite is observed for p-type materials. This type of electrical modification is utilized for gas sensing.

In the literature, sensitivity, response time, recovery time, optimum working temperature and lower limit of detection are reported as the main performance parameters of a sensor. Throughout the literature, sensor sensitivity (*S*) is defined in several different forms including *S* = *R_a_/R_g_, S* = *R_g_/R_a_, S* = Δ*R/R_g_* and *S* = Δ*R/R_a_*; where *R_a_* is the sensor resistance in ambient air, *R_g_* is the sensor resistance in the target gas, and ΔR = |*R_a_*−*R_g_*| [7,34,35]. In this literature review, the sensitivity values are reported as presented by the author. The formula used to calculate the sensitivity is also indicated. Response time is defined as the time required for a sensor to reach 90% of the total response of the signal such as resistance upon exposure to the target gas. Recovery time is defined as the time required for a sensor to return to 90% of the original baseline signal upon removal the target gas.

## 3. Fabrication of Gas Sensor with 1-D Nanostructures

1-D nanostructures used in the fabrication of gas sensors include metal-oxides in the form of nanorods, nanowires, nanofibers, nanotubes, nanobelts, nanoribbons, nanowhiskers, nanoneedles, nanopushpins, fibre-mats, urchin, lamellar and hierarchical dendrites. Nanorods, nanowire, nanofibers and nanotubes are rod shaped nanostructures having a diameter ranging from 1–200 nm. The aspect ratios (length divided by width) of nanorods and nanowires are 2–20 and greater than 20, respectively [36]. However, nanofibers have higher aspect ratio than nanowires. Nanotubes are basically hollow nanorods with a defined wall thickness. The definition of other nanostructures, such as nanobelts [22,37,38], nanoribbons [27], nanowhiskers [28], nanoneedles [29,39], nanopushpins [30], fibre-mats [21], urchin [31], lamellar [32] and hierarchical dendrites [20] can be found in the respective literatures. It is important to mention that the distinction between the different nanostructures is not always self evident and the terms are often used interchangeably from one reference to another.

**Figure 1.**
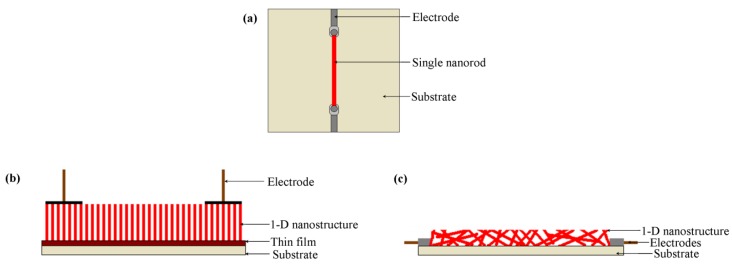
Schematics of sensor fabrication containing (**a**) a single nanostructure. (**b**) aligned nanostructures and (**c**) randomly distributed nanostructures.

These nanostructures can be arranged in different ways for the fabrication of a sensor. Figure 1 illustrates the predominant types of nanostructure arrangements and electrode attachment methods reported in literature. The nanostructure arrangements can be divided into three groups: (a) single nanostructure arrangement, (b) aligned arrangement and (c) random arrangement.

**Figure 2.**
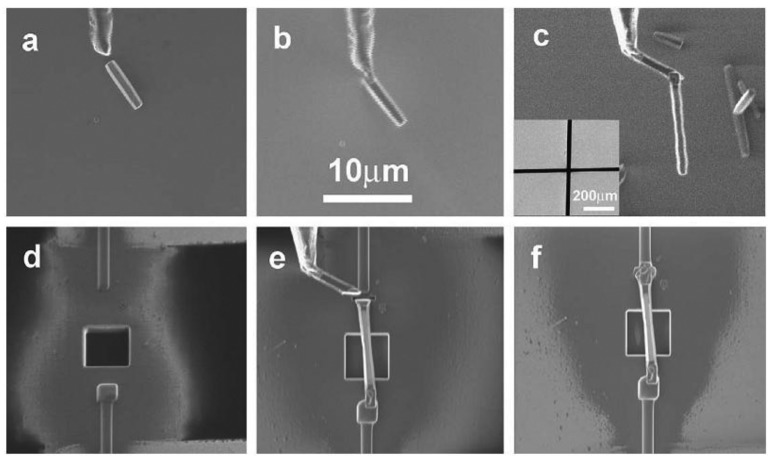
Scanning electron microscopy (SEM) images showing the steps of the *in-situ* lift-out fabrication procedure in the FIB/SEM system. (**a**) ZnO nanorod next to the FIB needle, (**b**) ZnO nanorod is picked-up by the needle, (**c**) selected ZnO nanorod is transferred for sensor fabrication, (**d**) a square hole cut on the glass, (**e**) positioning the ZnO nanorod over the hole and (**f**) single nanorod welded to both electrode/external connections as the final sensor [11].

Single nanofiber arrangement has been used by researchers for detecting a variety of gases such as H_2_ [11]. The nanostructure is often either a nanorod or a nanowire dependant on the diameter to length ratio [11,40]. Lupan *et al.* [11] developed an *in-situ* lift-out technique for arranging a single ZnO nanorod on a glass substrate to be used in H_2_ sensing applications. One single ZnO nanorod was attached to an electro-polished tungsten wire and positioned on a glass substrate containing a square hole for gas entrance. The nanorod was connected to the external electrodes as shown in Figure 2. Similarly, by using an *in-situ* lift-out technique by focused ion beam (FIB), single tripod and tetrapod gas sensors were developed from single ZnO nanorods by Lupan *et al.* [41,42] and Chai *et al.* [43]. Their technique obtained a 90% success rate for building prototypes of nano/micro-sensors based on individual nanoarchitectures from metal oxides.

For aligned nanostructure arrangements (Figure 1(b)), the nanostructure arrays are normally grown on a thin film. For example, Varghese *et al.* [44] developed a sensor device containing a TiO_2_ nanotube array which was adopted for exploring gas sensing properties. In this sensor, TiO_2_ nanotubes were grown from Ti foil by anodization [44]. A barrier layer also formed between the Ti foil and nanotubes during the process. Two spring-loaded parallel Pt pads (100 μm thickness) were used to contact the nanotubes electrically. A similar type of gas sensor was developed by Liao *et al.* [45] for detecting ethanol where ZnO nanorod arrays were sandwiched between a silicon substrate and an indium thin film. The indium thin film provided the Ohmic contact and a copper sheet was used as an electrode.

**Figure 3.**
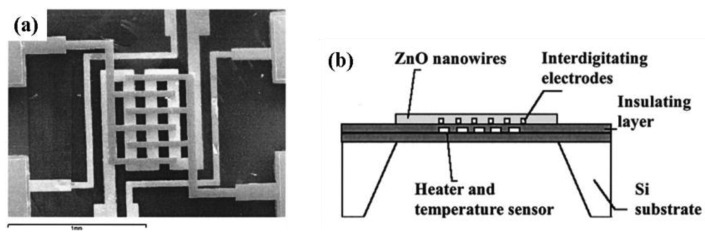
(**a**) Top view of the Pt interdigitated silicon substrate. (**b**) Schematic of the fabricated sensor structure [9].

Randomly distributed nanostructured sensors can have three variations: (i) nanostructures randomly distributed in the form of a film, (ii) randomly distributed nanostructures deposited on the circumference of a tube and (iii) randomly distributed nanostructures pressed into a tablet form. Wan *et al.* [9] used a flat interdigitated substrate where randomly distributed ZnO nanowires were dispersed in ethanol by ultrasonication directly coated onto a silicon-based interdigitated substrate by spin coating (Figures 1(c) and 3). This is common practice where the as-grown nanostructures are directly coated on the substrate through a standard technique such as spin coating [9]. Sometimes nanowire growth and attachment with the substrate is integrated with the device formation [46,47].

**Figure 4.**
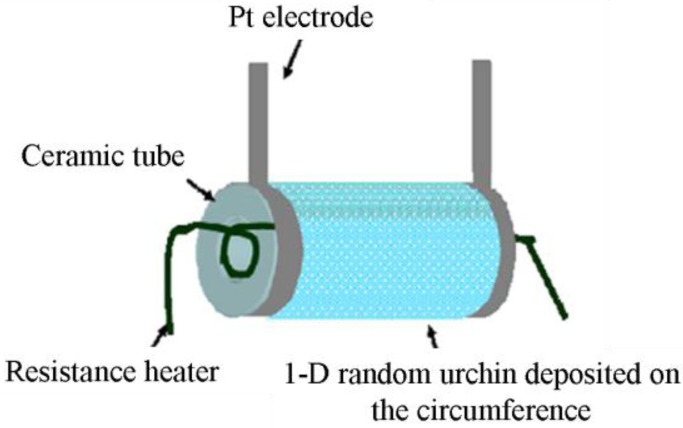
Schematic illustration of a tube-type 1-D nanostructured gas sensor [31].

Tube-type sensors are just one variation of film-type randomly distributed nanostructured sensors where the flat surface is shaped to a tube. This type of sensor consists of a ceramic tube which acts as a substrate as shown in Figure 4. Al_2_O_3_ is commonly used as the tube material. The surface of the tube is coated by the 1-D gas sensor materials. A variety of 1-D gas sensor materials with different morphology can be used on the surface of the ceramic tube. In Table 1, some reported tube-type gas sensors are listed with their dimensions and gas sensing materials. As an example, Hao *et al.* [31] fabricated a tube-type ceramic sensor for the detection of H_2_S. Porous 1-D α-Fe_2_O_3_ nano-urchins were mixed with terpineol to form a paste which was then coated uniformly onto the outside surface of an alumina tube having a diameter of 1 mm and length of 5 mm. A Ni-Cr alloy resistance heating coil was placed inside the tube to maintain the operating temperature. Pt wires were attached to gold electrodes for making the electrical contacts and finally connected to outside electronics for monitoring the resistance change. To improve performance, the gas sensors were heat treated at 300 °C for 10 days in air.

Randomly distributed nanostructures can also be used to fabricate tablet-type sensors. Zhou *et al.* [48] used such type of a sensor for ethanol gas. ZnO nanorods were formed in the shape of pellets under a 6 MPa pressure. The dimension of the pellets was 3 mm in thickness with a 5.3 cm^2^ area. High purity silver paste was used as an electrode and attached at the front and back side of the ZnO pellets by spin coating.

## 4. 1-D Nanostructured Materials for Gas Sensing

Over the last few years research on 1-D nanostructures for gas sensing applications has intensified because of their high surface-to-volume ratio, charge confinement ability and improved crystallinity. Several studies focused on the development of processing routes for the production of 1-D nanostructures for gas sensors. The yield, cost, complexity and quality of the materials obtained varied widely from process to process. A wide number of metal-oxides such as ZnO, SnO_2_, TiO_2_, In_2_O_3_, WO_x_, AgVO_3_, CdO, MoO_3_, CuO, TeO_2_ and Fe_2_O_3_ have been investigated for different target gases with varying degrees of success. In the following sections different types of 1-D nanostructured metal-oxides are discussed in terms of their growth, characterization and performance for gas sensing.

### 4.1. 1-D ZnO Nanostructured Gas Sensors

#### 4.1.1. Growth and Characterization of ZnO Nanostructures

The processing routes developed for the growth of 1-D ZnO nanostructures can be divided into three categories: (i) wet processing routes, (ii) solid-state processing routes and (iii) vapor-phase processing routes. Wet processing routes include hydrothermal and ultrasonic irradiation in an aqueous solution, while carbothermal reduction and solid-state chemical reaction are examples of solid-state processing routes for the production of ZnO nanostructure. Vapor-phase processing routes include molecular beam epitaxy (MBE), RF sputtering, aerosol, thermal evaporation, vapor-phase transport and chemical vapor deposition. Processing details for the growth of 1-D ZnO nanostructure are summarized in Table 2.

**Figure 5.**
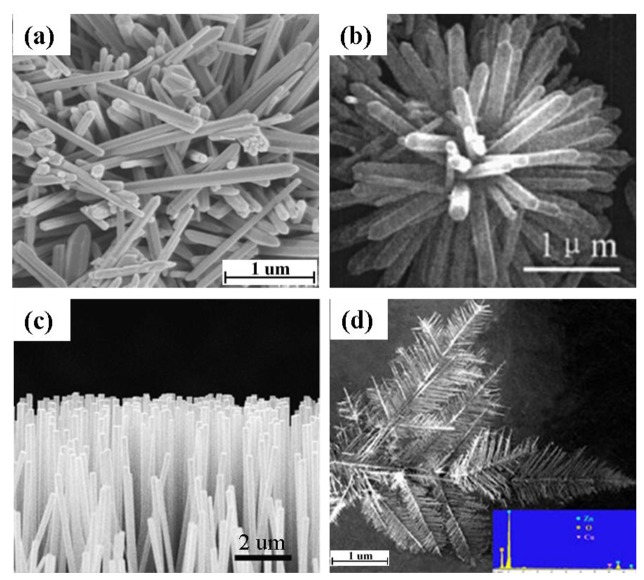
ZnO nanostructures. (**a**) Randomly distributed nanorods produced by hydrothermal process [48]. (**b**) Flowerlike nanorods produced by hydrothermal process [53]. (**c**) Vertically aligned nanorods produced by chemical vapor deposition process [55]. (**d**) Hierarchical dendrites produced by vapor-phase transport process [20].

Hydrothermal processing is the most widely employed method for the production of 1-D ZnO nanostructures due to its simplicity, low growth temperature, short growth interval, and ease of transfer of the product to other substrates [11]. Although the starting materials in a hydrothermal process vary widely, in all cases the main goal is to produce Zn(OH)_4_^2−^ ions which acts as a precursor for the fabrication of 1-D ZnO nanostructure (Table 2). The nanostructures obtained by hydrothermal process are mostly nanorods with different configurations such as vertically aligned [7], randomly distributed (Figure 5(a) and flowerlike (Figure 5(b)). It is seen that the addition of water in the hydrothermal process has a significant effect on the resulting nanostructure [52]. Addition of no or very low water content causes agglomeration and urchin type morphology of ZnO nanostructure. For obtaining ZnO nanorods, the addition of water is substantial. Recently, another simple wet processing route has been developed for the fabrication of vertically aligned ZnO nanorods by ultrasonic irradiation [12]. In this process, a Zn thin film was deposited on an interdigitated alumina substrate by RF sputtering technique. An ultrasonic wave was introduced to the sample after immersing the substrate in an aqueous solution containing Zn(NO_3_)_2_·6H_2_O and (CH_2_)_6_N_4_.

As mentioned previously, carbothermal reduction and solid-state chemical reaction are techniques used for producing ZnO nanowire in the solid state. Huang *et al.* [17] grew ZnO nanowire by a carbothermal reduction process on Au coated silicon substrates by heating a 1:1 mixture of ZnO and graphite powder at 900–925 °C under a constant flow of Ar gas. The as-grown nanowires had diameters of 80–120 nm with lengths of 10–20 μm. Cao *et al.* [18] produced ZnO nanorods by solid-state chemical reaction. The starting material for solid-state chemical reaction was ZnCl_2_ and NaOH with a molar ratio of 1:2 in presence of polyethylene glycol. The reaction involved the release of heat and evaporation of water vapor. It was suggested that in this process Zn(OH)_2_ precursor was formed by reacting ZnCl_2_ and NaOH, which subsequently decomposed into ZnO nanorods by an exothermic reaction. By adding Na_2_WO_4_-2H_2_O to the solution smaller nanorods were produced.

Vapor-phase processing has also been widely used for producing ZnO nanostructures. For example, Lupan *et al.* [55] grew vertically aligned ZnO nanowire (Figure 5(c)) by chemical vapor deposition (CVD) from Zn metal and O_2_/Ar flux. The Zn metal was evaporated at 670 °C in a quartz tube. The evaporated metal interacted with O_2_ at 650 °C on a Si substrate. The resulting nnaowires had a diameter of 100 nm with several micron length. Similarly, Wan *et al.* [19] grew ZnO nanowires on Zn pellets by thermal evaporation process by supplying Ar and O_2_ gas at 900 °C. Additionally, Zhang *et al.* [20] fabricated hierarchical ZnO dendrites (Figure 5(d) by a vapor-phase transport method at 930 °C from ZnO power in the presence of graphite and Cu catalyst. Comparing vapor-phase transport and thermal evaporation, no catalyst is required in the thermal evaporation process.

In the production of ZnO nanowires via the aerosol route, Zn vapor undergoes a fast expansion through a nozzle. Flower-mats and cauliflower type of nanostructures were obtained by aerosol route by supplying N_2_ gas on Zn powders at 500–750 °C [21]. ZnO produced by aerosol had a low yield compared to hydrothermal processing techniques resulting in only a 15% yield as determined by X-ray diffraction (XRD) analysis [21]. In contrast, the characteristics peak of Zn or other impurities could not be found in the nanostructure obtained by hydrothermal process [48].

Radio frequency (RF) sputtering is another vapor processing route where no metal catalyst is required for the production of ZnO nanostructures. ZnO nanobelts were produced on Pt interdigitated alumina substrates by RF sputtering technique as reported by Sadek *et al.* [22]. In the process of molecular beam epitaxy, O_3_/O_2_ plasma is discharged on Zn metal to produce ZnO nanorods on Au coated substrates [23].

Among the processing routes discussed, wet processing requires the lowest average temperature compared to solid-state processing and vapor-phase processing. The yield in wet processing is also high compared to other processing routes. However, wet processing mostly produces nanorods with different morphologies. In solid-state processing, the required temperature may be either room temperature (solid-state chemical reaction where heat is evolved during reaction) or in excess of 900 °C (carbothermal reduction). The obtained nanostructures in the solid-state processing consist of nanowires and nanorods with varying dimensions. Vapor-phase processing yields a verity of nanostructures including nanowires, nanorods, hierarchical dendrites, fiber-mats, cauliflower and nanobelts, though the yield is poor in some cases (e.g., aerosol route). The processing temperature in vapor-phase processing varies between 500–950 °C. A summary of these processing routes is presented in Table 2.

#### 4.1.2. Sensing Performance of ZnO 1-D Nanostructures

The performance of 1-D nanostructured ZnO sensors depends greatly on the processing techniques, surface morphology, sensor fabrication arrangements and operating temperature. Various target gases such as, C_2_H_5_OH, H_2_S, H_2_, NO_2_, CO, O_2_, HCHO, C_6_H_4_(CH_3_)_2_, NH_3_ and hydrocarbons have been tested to evaluate the performance of 1-D ZnO nanostructured sensors. Sensitivity, response time, recovery time, detection range, and optimum working temperature are the main performance parameters for gas sensors. Reported gas sensing properties for a variety of 1-D ZnO nanostructures for different gas species is summarized in Table 3.

In general, the sensitivity of 1-D ZnO nanostructure increases with an increase in the target gas concentration. Depending on the processing route, ZnO nanostructures can be obtained in different surface states, size and morphology. Changes in these parameters can result in variations in gas sensing properties [18]. For example, the surface morphology of 1-D ZnO nanostructures greatly affects the performance of the sensor. Wang *et al.* [5] showed that the surface roughness improves the sensitivity of ZnO nanorods. It was observed that the addition of surface smoothening agents such as sodiumdodecyl sulfate during nanorod fabrication resulted in decreased sensitivity. A rougher surface exhibits higher sensitivity because it provides more active sites for oxygen and reducing gases on the surface of the sensor material. Also, nanostructures having smaller size have higher surface area resulting in higher gas sensitivity [18,52].

**Figure 6.**
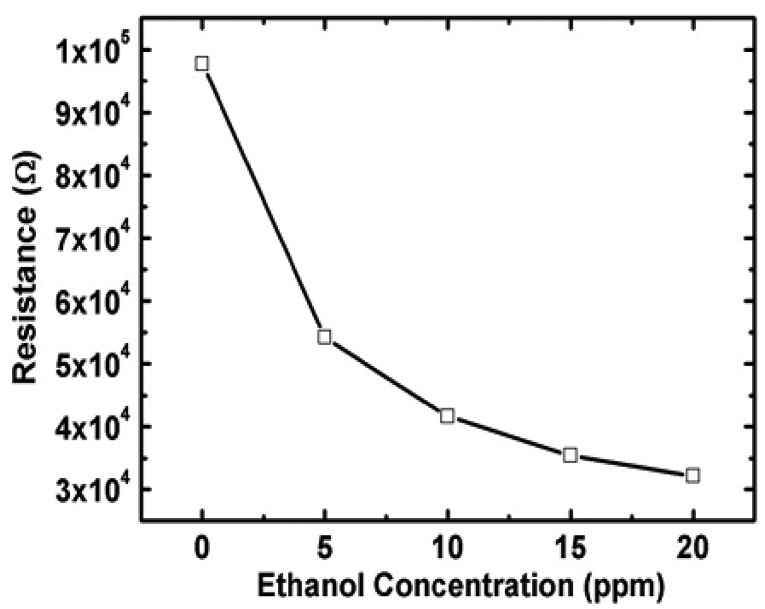
Resistivity of n-type ZnO sensor is decreased when exposed to reducing ethanol environment [48].

It is seen from Table 3 that the sensitivity of ZnO nanorods towards ethanol is high compared with other target gases. The resistivity of an n-type ZnO sensor is decreased when exposed to reducing ethanol environment as it can be seen in Figure 6 [48]. Thus far different types of nanostructures including nanowires (laterally grown, randomly distributed) and nanorods (flowerlike, bushlike, vertically aligned) were examined to evaluate their performance towards ethanol gas. It was seen that laterally grown ZnO nanowires had higher sensitivity than randomly oriented ZnO nanowires [9,47]. It was also seen that flowerlike [53] and bushlike [57] nanorod assemblies had relatively low response towards ethanol compared to the nanowire morphology. Among all described nanostructure assemblies, vertically aligned nanorods showed the highest sensitivity towards ethanol gas at a temperature of 300 °C and a concentration of 100 ppm [7]. In addition to resistance, other parameters such as capacitance also changed when 1-D ZnO nanostructure were exposed to a reducing environment. One such experiment was carried out by Zhou *et al.* [48] for the detection of ethanol using ZnO nanorods. It was seen that the capacitance increased and resistance decreased with an increase in ethanol concentration at low frequencies (10^2^ to 10^4^ Hz). At high frequency, ranging from 10^4^ to 10^6^ Hz, the capacitance and resistance changes were negligible. Data on the response and recovery time of ZnO nanostructures for ethanol gas sensing are not available in most literature. Based on the limited available data, the response and recovery time of ZnO nanorod is 3 min and 4 min, respectively in an ethanol environment [48]. Another important parameter is the optimum operating temperature for which very limited data is available. Wang *et al.* [5] measured the optimum operating temperature of ZnO nanorods for ethanol sensing and found the response improved at higher temperature (350 °C). Higher bonding energies of H-CH_2_ (473 KJ/mol), H-OC_2_H_5_ (436 KJ/mol) and H-CH (452 KJ/mol) in C_2_H_5_OH led to the increase in optimum operating temperature [58].

ZnO nanostructures also show higher sensitivity to H_2_S compared to other target gases such as H_2_, NO_2_ and hydrocarbons. Hierarchical dendrites of ZnO showed increased sensitivity towards H_2_S compared to NH_3_, H_2_ and NO_2_ in dry air at 30 °C [20]. The sensitivity of hierarchical dendrites of ZnO towards H_2_S is 26.4 for 500 ppm gas concentration at 30 °C. Other forms of 1-D ZnO nanostructures such as ZnO nanorods have lower reported sensitivities than ZnO hierarchical dendrites [20,45]. The response and recovery time of hierarchical ZnO dendrites are reported to be 15–20 s and 30–50 s, respectively [20]. The optimum operating temperature for ZnO nanorods is 25–200 °C for H_2_S gas sensing which is lower compared with ethanol sensing [5]. The bonding energy of H-SH in H_2_S is 381 KJ/mol [58], which makes it relatively easy to break the bond of H_2_S at low temperature.

Many reports in the literature agree that ZnO nanostructures have poor sensitivity towards H_2_ gas [5,57]. However, it has also been seen that single ZnO nanorod and single ZnO nanowire sensor assemblies can detect H_2_ gas at room temperature in presence of dry air [11,59]. But at room temperature, the sensitivity of ZnO nanowires is only 3 and 4 for 100 ppm and 200 ppm H_2_, respectively [11,59]. The addition of catalysts was found to increase the sensitivity of ZnO nanorods. Wang *et al.* [23] coated ZnO nanorods with Pd and found the response increased by approximately a factor of 5 relative to an uncoated nanostructure. Catalytic dissociation of H_2_ to atomic hydrogen by Pd is a possible reason for the increased sensitivity. Out of the nanostructures discussed, ZnO nanobelts showed the highest response of 14.3 for 1% H_2_ concentration at the optimum working temperature of 385 °C [22]. It is important to note that most of the research done for H_2_ sensing was performed at room temperature. However, Sadek *et al.* [22] found that ZnO nanostructures showed a considerable sensitivity for H_2_ gas at 385 °C. It may be the case that the low response of ZnO nanostructures found in the previous literature is due to the low working temperature. It was found that the recovery time of Pd coated ZnO nanorods were <20 s, whereas the recovery time for ZnO nanorod and nanobelt was 50–90 s and 336 s, respectively [11,22,23]. The response time for single ZnO nanorod sensor was quite short and found to be only 30–40 s [11].

1-D ZnO nanostructures also displayed a good response toward oxidizing NO_2_ gas detection. The resistance of the sensor increased when exposed to NO_2_ environment [52]. A ZnO nanowire floated on SiO_2_ substrate was able to detect NO_2_ gas down to 0.5 ppm level at 225 °C [54]. Additionally, an array type of sensor containing vertically aligned ZnO nanorods detected NO_2_ gas at the 10 ppb level [12]. The sensitivity of ZnO fibre-mats was reported to be more than 100 towards NO_2_ at room temperature [21]. The fibre-mats structure had higher response by an order of magnitude compared with the cauliflower structure. The difference between the sensing properties of these two structures can be ascribed to the differences in their morphologies, since the available surface for reaction is higher in fibre-mats compared to cauliflower structure. It was found that the response and recovery time of 1-D ZnO sensors varied from few tenths of seconds to few minutes.

1-D ZnO nanostructures have been reported to have a very poor response to CO, O_2_, and CH_4_ gases at room temperature [11]. Hsueh *et al.* [47] measured the sensitivity of ZnO nanowires having different diameters and length for CO sensing. It was seen that thinner and taller ZnO nanowires could detect CO gas more efficiently compared to wider and shorter nanowires. For example, at 320 °C, ZnO nanowires having diameter of 50–70 nm and length of 5.4 μm had a response of 57% at 500 ppm CO concentration. This variation in the result from Hsueh *et al.* [47] and Lupan *et al.* [11] could be attributed to the difference in detection temperature used in the study. Hsueh *et al.* [47] measured the sensitivity towards CO at 320 °C, whereas Lupan *et al.* [11] measured the sensitivity at room temperature.

Sensitivity of ZnO nanorods towards methanol (HCHO) and xylene (C_6_H_4_(CH_3_)_2_) was investigated by Cao *et al.* [18]. ZnO nanorods exhibited good sensitivity to HCHO and C_6_H_4_(CH_3_)_2_ at low working temperatures. Nanorods having smaller dimensions (length: 100 nm, diameter: 20–40 nm) exhibited higher sensitivity compared to nanorods having larger dimensions (length: 200 nm, diameter: 40–60 nm). It was claimed that the higher sensitivity in the smaller nanorods was due to the increased surface area as seen in Table 3.

ZnO nanostructures also show a good response towards hydrocarbons such as methane [5] and propane [11,22]. The optimum working temperature evaluated for propane was 370 °C with a response and recovery time of 72 s and 252 s, respectively. The sensitivity towards propane was not as high as other target gases but still, the results showed a promising response for industrial applications. The response of ZnO nanobelts towards 1% propane at 370 °C was 0.17. The response of ZnO nanorods towards methane was further lower and found to be only 0.002 at room temperature [11].

Gas sensing properties of one-dimensional ZnO nanorods exhibit improved response and stability than those of ZnO nanoparticles [61]. Previously, it was demonstrated that uniform ZnO nanorods can be used to improve the response of ZnO based gas sensors to H_2_ gas [23,61]. However, the Pd-coated ZnO nanowires gas sensors reported by Wang *et al.* showed a higher H_2_ sensitivity (4.2%) and fast response and recovery time at concentrations up to 500 ppm at room temperature [62]. In general, it can be said that 1-D ZnO nanostructures can detect ethanol and H_2_S gas most efficiently. The sensitivity of 1-D ZnO nanostructures towards other gases such as H_2_, NO_2_, CO, O_2_, hydrocarbons is comparatively low without additional functionalization by catalyst doping. The response and recovery times show a direct dependence on the target gas. The performance of the sensors depends greatly on the morphology of 1-D ZnO nanostructures and the operating temperature used.

### 4.2. 1-D SnO_2_ Nanostructured Gas Sensors

#### 4.2.1. Growth and characterization of SnO_2_ Nanostructures

The processing routes developed for the growth of 1-D SnO_2_ nanostructures can be divided into four categories: (i) wet processing routes, (ii) molten-state processing routes (iii) solid-state processing routes and (iv) vapor-phase processing routes. The wet processing routes include hydrothermal and electrospinning, while the molten-state processing routes involve the use of a molten salt solution. Nanocarving and direct oxidation represent solid-state processes whereas thermal evaporation is used in the vapor-phase processing route. A hybrid route was also developed by combining electrospinning process with pulsed laser deposition. The processing methods for the growth of 1-D SnO_2_ nanostructure are summarized in Table 4.

Few reports of the production of SnO_2_ nanostructures by hydrothermal methods have been reported as compared to ZnO. However, Lupan *et al.* [63] reported an inexpensive and rapid fabrication technique for rutile SnO_2_ nanowires/nanoneedles at a low temperature by a hydrothermal method without the use of seeds, templates or surfactants. A solution containing SnCl_4_·5H_2_O, NH_4_(OH) was employed for the growth of SnO_2_ nanowires/nanoneedles at 95–98 °C on Si/SiO_2_ substrates. Individual nanowires can be easily transferred to other substrates for fabricating single nanowire ultrasensitive sensors [11]. The resulting nanowires/nanoneedles have a diameter of approximately 100 nm with lengths of 10–20 μm. The morphology, dimension and aspect ratio of nanowires are a function of growth time, temperature and Sn^4+^/OH^−^ ratio in solution. Thinner nanowires can be produced by decreasing the concentration of SnCl_4_ in solution. When the ratio between SnCl_4_ and NH_4_OH was as high as 1:20, long tetragonal square-based nanowires were obtained. Experimental results showed that the molar ratio of 1:20 made the hydrolysis occur rapidly due to a higher quantity of nuclei. By further increasing the ratio above 1:30 no nanowires were formed. Similarly, Shi *et al.* [64] produced SnO_2_ nanorods by hydrothermal process and then loaded the nanorods with La_2_O_3_ by simple chemical method. The SnO_2_ nanorods were synthesized from the precursors SnCl_4_-5H_2_O and NaOH at 190 °C in an alcohol/water solution. La_2_O_3_ was then loaded on the SnO_2_ nanorods by dispersing the nanorods in alcohol followed by the addition of La(NO_3_)_3_-6H_2_O solution.

**Figure 7.**
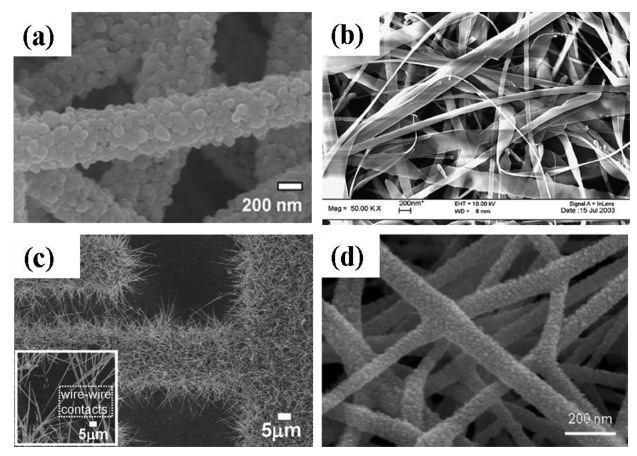
Scanning electron microscopy (SEM) images of (**a**) SnO_2_ nanofibers produced by electrospinning after heating at 600 °C for 2 h [56]. (**b**) SnO_2_ nanoribbons synthesized by direct oxidization [27]. (**c**) On-chip fabrication of SnO_2_ nanowires grown on Au deposited Pt interdigitated substrate by thermal evaporation [10]. (**d**) SnO_2_-ZnO hybrid nanofiber by electrospinning [67].

Qi *et al.* [13] grew SnO_2_ nanofibers by the electrospinning technique. In this process, SnCl_2_ was mixed with *N,N*-dimethylformamide (DMF) and ethanol subsequently adding poly(vinyl pyrrolidone) (PVP) under vigorous stirring. Then the mixture was loaded into a glass syringe with a 10 kV power supply between the cathode and anode. The conversion of SnCl_2_ to SnO_2_ and the removal of PVP were achieved by calcining at 600 °C for 5 h in air. Choi *et al.* [65] also produced Pd doped SnO_2_ hollow nanofibers by single capillary electrospinning process. In this procedure SnCl_2_-2H_2_O was dissolved in mixed solvents of ethanol and *N,N*-dimethylformamide followed by stirring and addition of PVP. After stirring for 10 h, a clear solution was obtained and used for the preparation of undoped SnO_2_ nanofibers. For the fabrication of Pd-doped SnO_2_ nanofibers, PdCl_2_ was added to the solution. The solution was loaded in a plastic syringe and electrospun by applying 20 kV at an electrode distance of 10 cm. The as-spun fibers were heat treated at 600 °C for 2 h to convert into undoped or Pd-doped SnO_2_ nanofibers. Dong *et al.* [56] also developed Pt doped SnO_2_ nanofibers by electrospinning with a similar procedure as that reported by Choi *et al.* and as seen in Figure 7(a) [65]. After synthesis of the SnO_2_ nanofibers, PtCl_4_ was added to the solution and loaded in a plastic syringe followed by electrospining at a voltage of 20 kV with 10 cm electrode distance. The as-spun fibers were heat treated at 600 °C for 2 h.

ZnO nanorods were also prepared by molten-salt method where SnO_2_ powder was mixed with NaCl and a nonionic surfactant [16]. The mixture was heated in a porcelain crucible at 800 °C in an electric furnace followed by cooling, washing in distilled water, filtering and drying. It was claimed that addition of the nonionic surfactant formed a shell surrounding the SnO_2_ particles to prevent agglomeration and ensured uniform nanorods.

A novel route was developed by Carney *et al.* [66] for the production of SnO_2_ by a vapor-assisted growth process. In this procedure, SnO_2_ powder was mixed with CoO (solid-state sintering aid) and compacted to a 0.64 cm disk under 880 MPa pressure followed by sintering at 1,500 °C. The disk was coated with Au nanoparticles and exposed to humid 5%H_2_ with balance N_2_ at 700 to 800 °C. The resulting nanofibers had 100–200 nm diameters. Increasing the exposure time to the gas mixture resulted in an increase in the average nanofiber length. It was found by further investigations that the presence of Au nanoparticles was essential to assist the growth of nanofibers. Direct oxidation is another solid-state processing route where SnO_2_ nanoribbons (Figure 7(b)) were grown at 810 °C from Sn powders in the presence of Ar gas flow [27]. To modify the surface of SnO_2_ nanoribbons, CuO was introduced to the nanoribbons by mixing SnO_2_ and CuO in distilled water.

Ying *et al.* [28] developed a process route to synthesize SnO_2_ nanowhiskers by thermal evaporation on Au coated Si substrate. Sn powder of 99.9% purity was heated at 800 °C on an alumina boat with a constant flow of 99% N_2_ and 1% O_2_. The resultant nanowhiskers had a rectangular cross-section with diameters of 50–200 nm and lengths up to tens of micrometers. Similarly, Thong *et al.* [10] also developed SnO_2_ nanowires on Au deposited interdigitated Pt substrate by thermal evaporation process (Figure 7(c)). In this procedure, Sn powder was heated to 800 °C on alumina boat with a constant supply of O_2_ (0.3 sccm). The substrate was kept 1.5 cm away from the source. The pressure inside the tube was maintained at ∼2 Torr and the growth time was varied from 15 to 60 min. With increasing growth time from 15–60 min, the length of the nanowires increased from 40–85 nm. It was also observed that SnO_2_ nanowires only grew in the substrate area where the Au catalyst was deposited. A two step thermal evaporation procedure was used to grow hierarchical SnO_2_ nanowires on Au deposited interdigitated Pt substrate by thermal evaporation process [68]. In the first step, SnO_2_ nanowires were grown at 980 °C on the substrates using SnO powder and oxygen supply inside a quartz tube. The second step was carried out at 800 °C with Sn powder and oxygen as the source. These two steps done in series produced hierarchical SnO_2_ nanowires. The O_2_ flow rate inside the quartz tube was maintained at 0.3–0.5 sccm with pressure of ∼2–5 Torr. The SnO_2_ nanobelts were deposited on an alumina plate by thermal evaporation process at 1,000 °C by using SnO powder and Ar gas at 300 Torr pressure and without using any catalyst [37]. The deposited SnO_2_ nanobelts were retrieved from the alumina substrate and separated into individual nanobelts in an isopropyl alcohol solution via ultrasonic agitation.

A hybrid process was also reported for the production of mixed SnO_2_-ZnO composite oxide nanostructures [67]. For this preparation, Zn(CH_3_COO)·2H_2_O was mixed with poly(4-vinylphenol) and stirred for 3 h at 60 °C followed by addition of ethanol. The solution was then loaded into a plastic syringe with a voltage supply of 7 kV. The substrate temperature was maintained at 80 °C. The as-prepared ZnO nanofibers were collected on Pt interdigitated SiO_2_/Si substrate and calcined at 600 °C. The SnO_2_ was deposited on the ZnO nanofibers using pulsed laser deposition (PLD) method with KrF excimer laser (λ = 248 nm). A scanning electron microscopy (SEM) micrograph of SnO_2_-ZnO nanofibers is shown in Figure 7(d).

Among the synthesis methods, thermal evaporation and electrospinning are the most commonly employed methods for the production of SnO_2_ nanostructures. The nanostructures obtained by hydrothermal and electrospinning processes are nanorods and nanofibers, respectively. The processing temperature in the molten-salt processing route is 800 °C and produced nanorods. However, presence of Au catalyst is essential during vapor-assisted growth process for the production of SnO_2_ nanofibers. In the thermal evaporation process, heat (800–980 °C) and pressure are involved and a variety of nanostructures could be obtained including nanowires (normal, hierarchical), nanobelts and nanowhiskers. In this synthesis method, SnO_2_ nanostructures grow only in the presence of Au catalyst. A summary of the various processing routes is presented in Table 4.

#### 4.2.2. Sensing Performance of SnO_2_ 1-D Nanostructures

In the reported literatures, the sensitivity of SnO_2_ nanostructures was evaluated for different target gases such as ethanol, H_2_S, H_2_, NH_3_, liquefied petroleum gas (LPG), toluene, acetone and triethylamine. The morphology of the nanostructures employed for sensing included nanorods (normal, flowerlike), nanowires (normal, hierarchical), nanofibers, nanobelts and nanowhiskers. Sensitivity, response time, recovery time and optimum detection temperature were considered to evaluate the sensing performance.

Ying *et al.* [28] synthesized SnO_2_ nanowhiskers by thermal evaporation for ethanol sensing. The sensitivity of SnO_2_ nanowhiskers was 23 upon exposure to 50 ppm ethanol at 300 °C. The recovery time was about 10 min. Nanorods with a flowerlike morphology developed by Shi *et al.* [64] had a response of 45.1 at 200 °C for 100 ppm ethanol concentration. The response was further increased by developing La_2_O_3_ loaded SnO_2_ nanorods with a flowerlike morphology by Shi *et al.* [64]. It was seen that the sensitivity of 5 wt% La_2_O_3_ loaded SnO_2_ nanorods had a response of 213 whereas without loading had only 45.1 at 200 °C for 100 ppm ethanol concentration. The increased sensitivity with the loading of 5 wt% La_2_O_3_ on SnO_2_ nanorods was explained by the basic nature of La_2_O_3_. The presence of La_2_O_3_ reduces the acidic sites and leads to increase in the dehydrogenation process [69,70]. As a result, many more CH_3_CH_2_OH molecules convert to CH_3_CHO due to the presence of La_2_O_3_ which creates a favorable condition to convert to CO_2_ and H_2_O from the thermodynamic point of view [71]. Choi *et al.* [65] showed significantly different responses towards C_2_H_5_OH with Pd doping on SnO_2_ hollow nanofibers. Selective detection of C_2_H_5_OH was observed with the doping of Pd on SnO_2_ hollow nanofibers. In 0.4 wt% Pd-doped SnO_2_ hollow nanofibers, the response to 100 ppm C_2_H_5_OH was 1,020.6 at 330 °C, whereas CH_4_, CO, H_2_ had very negligible responses. However, the response of C_2_H_5_OH decreased dramatically as the sensor temperature was increased from 330 to 440 °C, while response to CH_4_ and H_2_ was increased or only varied only slightly. Therefore, the selective detection of H_2_ and/or CH_4_ was optimized at 440 °C with the minimum interference to C_2_H_5_OH. The selective gas sensing was explained in terms of the different catalytic oxidation activities of the analyzed gases as a function of sensor temperature and Pd doping concentration. The response time was evaluated to be <10 s for this sensor but the recovery time was higher at about 503 s for 100 ppm C_2_H_5_OH at 385 °C. The slow recovery was explained by the sluggish surface reactions of adsorption, dissociation, and ionization of oxygen. It was found that with increase in temperature the recovery time decreased.

The performance of SnO_2_ nanofibers was evaluated for H_2_S gas and the response to 20 ppm concentration was found to be 121 at 300 °C [56]. It was also observed that the response tended to decrease with an increase in temperature from 300 to 500 °C. For SnO_2_ nanofibers, the response time varied between 2 and 7 s and the recovery time varied between 267 and 281 s. However, the sensitivity of the SnO_2_ sensors could be further increased by Pt doping [56]. The doping of 0.08 wt% of Pt on SnO_2_ nanofibers produced a response of 5,100 for 20 ppm H_2_S gas at 300 °C. The response time of Pt doped SnO_2_ nanofibers was found to be faster (1 s) compared to undoped SnO_2_ nanofibers (2–7 s). The surface modification due to the Pt doping increased the resistance of the nanofibers which indicated higher grain barriers. Increased resistance in the grain barrier might be due to a higher oxygen adsorption introduced by the presence of Pt, or may be directly related to the presence of a Pt catalyst at the grain surface [72,73]. SnO_2_ nanoribbons in presence of CuO nanoparticles showed a sensitivity of 18,000 towards H_2_S gas at 50 °C [27]. Presence of CuO nanoparticles formed n-p junction in the nanoribbons network. However, existence of H_2_S gas forms a thin CuS layer on CuO nanoparticles, which is a good conductor. As a result, the n-p hetero-junction was converted into a Schottky barrier which induced a remarkable change in the sensitivity.

Fields *et al.* [74] developed a SnO_2_ nanobelt-based sensor for H_2_ detection. It was found that the sensitivity of SnO_2_ nanobelts at 25 °C was 60% and remained nearly constant up to 80 °C for 2% H_2_ concentration. It was found that both the response and recovery times were about 220 s at 25 °C. It was also found that when the temperature was increased to 80 °C, the response time decreased to about 60 s, while the recovery time increased to about 500 s. The relatively long response time is believed to be caused by the low chemical reaction rate. It is likely that the response can be improved by coating the nanobelt surface with a catalyst such as Pd or Pt in order to produce a practical room-temperature H_2_ sensor.

The length of SnO_2_ nanowires had an impact on the sensor's performance for detecting NH_3_. Longer nanowires showed higher sensitivity toward NH_3_ gas compared to shorter nanowires. The response to 1,000 ppm NH_3_ at an operating temperature of 200 °C varied from 3 to17 with varying the nanowire length from 40–85 nm [10]. It was observed that hierarchical nanowires showed higher response towards NH_3_ compared to the normal nanowires. For 1,000 ppm NH_3_ concentration at 200 °C, the response of hierarchical nanowires was found to be 21.7 [68], whereas the response of normal nanowires was 11 [10].

Like NH_3_, the response of SnO_2_ nanowires towards liquefied petroleum gas (LPG) depends on the length of the nanowire. Longer nanowires exhibit an increased response compared to shorter nanowires. In the experiments of Thong *et al.* [10], the response of SnO_2_ nanowires increased from 1.5 to 21.8 when the length of the nanowire was increased from 40 to 85 nm at 350 °C for 2,000 ppm LPG. The optimum working temperature was determined to be 350 °C with a response and recovery time of less than 10 s. Comparing the response of hierarchical nanowires with normal nanowires, it was seen that the sensitivity towards LPG was increased three times at the optimal operating temperature [68]. It was found that the response of normal SnO_2_ nanowires (length 60 nm) for 2,000 ppm LPG was 5.8 [10], whereas the response of hierarchical SnO_2_ nanowires was 20.4 at 350 °C [68].

Qi *et al.* [13] showed that SnO_2_ nanofibers can detect toluene at 350 °C with response and recovery times of 1 s and 5 s respectively. The optimum working temperature was 350 °C with sensitivity for 1,000 ppm toluene of 19. The sensitivity of acetone and triethylamine was studied by Wang *et al.* [16] by using single crystalline SnO_2_ nanorods. By adding an additional surfactant the sensitivity towards gases containing N or O atoms, like triethylamine, was improved.

Composite nanofibers of SnO_2_ and ZnO were exposed to various NO_2_ concentrations by Park *et al.* [67]. The optimum sensitivity of the SnO_2_-ZnO composite nanofiber was found to be between 180–200 °C operating temperatures. The sensitivity for 3.2 ppm NO_2_ was 105 at 200 °C. High sensitivity towards NO_2_ forthe SnO_2_-ZnO composite nanofibers was reportedly due to two factors: the increased adsorption due to nanocrystalline SnO_2_ coating and the charge transfer occurring between SnO_2_ and ZnO.

From the review of reported literature, it can be surmised that SnO_2_ nanostructure-based sensors were developed with reasonable success for detecting a range of gases including ethanol, H_2_S, H_2_, NH_3_, liquefied petroleum gas (LPG), toluene, acetone, NO_2_ and triethylamine. However, the sensitivity and selectivity can be further improved by doping (Pd and Pt), adding nanoparticles (CuO), loading (La_2_O_3_), and morphological modifications (hierarchical nanowires). Additionally, preparation of composite nanostructures (SnO_2_-ZnO nanofiber) also improves the sensitivity and selectivity of the sensors. Unlike ZnO nanostructures, the response and recovery times of the SnO_2_ nanostructures show a strong dependence on the operating temperature. The optimum operating temperature is of vital importance since by simply adjusting the operating temperature, SnO_2_ sensors can be used for selective gas sensing. A summary of SnO_2_ nano-structured sensor performance is presented in Table 5.

### 4.3. 1-D TiO_2_ Nanostructured Gas Sensors

#### 4.3.1. Growth and Characterization of TiO_2_ Nanostructures

The processing routes for the synthesis of 1-D TiO_2_ nanostructures can be divided into two groups: (i) wet processing routes and (ii) solid-state etching. Most commonly wet processing route is employed for the synthesis of 1-D TiO_2_ nanostructure. The wet processing route includes hydrothermal, electrospinning and anodization. Nanocarving by H_2_ gas, UV lithography and dry plasma etching fall under solid-state etching process. Depending on the processing routes and conditions different surface morphologies such as nanotube arrays, branched nanotubes, coated nanotubes, nanoparticle added nanotubes, nanobelts, nanofibers and nanowires of TiO_2_ can be obtained. The crystal structure also can be changed by annealing. The processing details for the growth of 1-D TiO_2_ nanostructures are summarized in Table 6.

**Figure 8.**
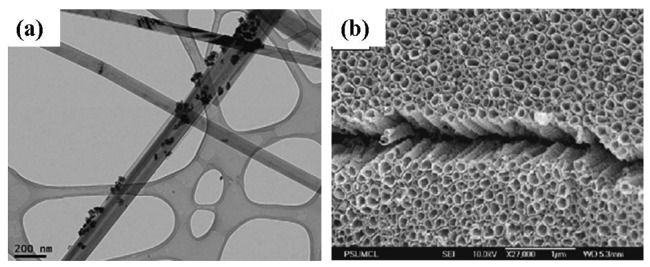
(**a**) Transmission electron microscopy (TEM) image of Pt nanoparticles added TiO_2_ nanotubes [75]. (**b**) Scanning electron microscopy (SEM) micrograph of the top view of the TiO_2_ nanotube array grown by anodization process [79].

Rout *et al.* [59] synthesized TiO_2_ nanowires by hydrothermal process by using TiCl_3_ solution in HCl and saturated NaCl. The mixture was put in a Teflon-lined autoclave and heated at 200 °C for 2 h. The product obtained after cooling the autoclave to room temperature was washed with deionized water and alcohol followed by drying in vacuum. The resulting nanostructures had diameters of 20–80 nm and lengths of 100–800 nm. The crystal structure was found to be rutile. Additionally, Han *et al.* [75] synthesized Pd and Pt nanoparticel-TiO_2_ nanotubes by hydrothermal processing. A transmission electron microscopy (TEM) image of Pt nanoparticles added TiO_2_ nanotubes is shown in Figure 8(a). Commercial anatase TiO_2_ powder and PdCl_2_ or H_2_PtCl_6_ were dispersed in an aqueous solution of NaOH and charged into a Teflon-lined autoclave. The autoclave was heated at 150 °C for 12 h. The precipitates were separated by filtration and washed with dilute HCl and de-ionized water. The synthesized Pd and Pt nanoparticle-TiO_2_ nanotubes were dried at 120 °C in an oven. The resulting nanotubes were 100 nm in diameter with a lepidocrocite-type phase of titanate. Hu *et al.* [38] prepared TiO_2_ nanobelts via an alkaline hydrothermal process by using commercial TiO_2_ powders, NaOH, HCl, and deionized water. The obtained H_2_Ti_3_O_7_ nanobelts were annealed at 600 °C for 1 h to obtain crystalline TiO_2_ nanobelts. The surface of the TiO_2_ nanobelts was coarsened by adding H_2_SO_4_ into H_2_Ti_3_O_7_ aqueous solution under magnetic stirring followed by heating at 100 °C for 12 h. The powder was washed and annealed at 600 °C for 1 h to obtain surface-coarsened TiO_2_ nanobelts. For the preparation of Ag nanoparticle-TiO_2_ nanobelts and surface coarsened Ag nanoparticel-TiO_2_ nanobelts, the hydrothermal process was combined with a photocatalytic reduction process [38]. The as-prepared TiO_2_ nanobelts obtained by hydrothermal route were dispersed into AgNO_3_ and ethanol solution. The solution was illuminated with a 20 W ultraviolet lamp under magnetic agitation. The obtained phase of TiO_2_ nanobelt was anatase.

Landau *et al.* [76] synthesized TiO_2_ nanofibers by electrospinning. The electrospun solution comprised of poly(vinyl acetate) (PVA), dimethylformamide (DMF), titanium (IV) propoxide, and acetic acid in an electric field of 1.5 KV/cm. The solution was electrospun at room temperature on wax paper or Si wafer rotating at 100 rpm. It was seen that the morphology of the nanofiber depended on the concentration of PVA. At low concentration (≤2 wt%) the network comprised of small beads interconnected by thin fibers whereas at higher concentration (≥5 wt%) the beads disappeared and the fiber became continuous and homogeneous. The diameter of the nanofibers was seen to increase from 120 nm to 850 nm with an increase in the polymer concentration from 5 wt% to 12 wt%.

To prepare Cu-doped TiO_2_ nanofibers by electrospinning tetrabutyl titanate was mixed with acetic acid and ethanol under vigorous stirring for 10 min [50]. Subsequently, this solution was added to ethanol containing PVP and CuCl_2_-2H_2_O under vigorous stirring for 30 min. Then, the mixture was loaded into a glass syringe and connected to a high-voltage power supply of 12 kV over a distance of 20 cm between the electrodes. The conversion of tetrabutyl titanate to TiO_2_ and the complete removal of PVP were achieved by calcining at 500 °C for 3 h in air. It was found from the XRD analysis that the crystallographic phases were 20% anatase and 80% rutile with a nanofiber diameter of 80 nm.

Varghese *et al.* [44] grew TiO_2_ nanotubes on titanium foil by anodization. In this process a platinum foil was used as a cathode and titanium foil as an anode at an anodization potential of 12 V and 20 V between the electrodes. The electrolyte medium consisted of 0.5% hydrofluoric acid in water. The samples were then annealed at 500 °C in pure oxygen for 6 h. From field emission scanning election microcopy (FESEM) it was seen that the nanotubes were approximately 400 nm in length with a 46–76 nm diameter. A barrier layer with a thickness of 50 nm was formed in between the nanotubes and foil. It was also seen that with an increase in the anodization voltage, the pore diameter of the nanotube increased. Nanotubes fabricated using 20 V had an average pore diameter of 76 nm with a wall thickness of 27 nm. Additionally, samples anodized at 12 V were found to have an average pore diameter of 46 nm with a wall thickness 17 nm. Both anatase and rutile phases of titania were found to be present in the samples. Anatase concentrated on the walls of the nanotubes and rutile in the barrier layer [77]. Nanotubes were found to be mechanically stable (intact) up to 580 °C. Above this temperature the nanotubes collapsed due to grain growth leading to protrusions. Similarly, Lu *et al.* [78] also synthesized TiO_2_ nanotube arrays by anodization of a 250 μm thick titanium foil. The titanium foil was used as an anode and a platinum foil was used as a cathode under a constant potential of 20 V. The electrolyte for the synthesis consisted of NH_4_F and (NH_4_)_2_SO_4_ in deionized water. The anodic oxidation process was conducted at room temperature for 2 h. The as prepared amorphous TiO_2_ nanotube arrays were annealed at 450 °C in air for 2 h to obtain anatase TiO_2_. The resulting nanostructure had an outer and inner diameter of 150 nm and 110 nm, respectively with length of approximately 2.3 μm. The nanotube dimension could be varied in the anodization process by changing both the pH of the electrolyte and the electrode voltage [79]. In the work of Paulose *et al.* [79] nanotube arrays were prepared by anodization of 250 μm thick titanium foils in an electrolyte containing sodium hydrogen sulfate monohydrate, potassium fluoride and sodium citrate tribasic dihydrate (Figure 8(b)). The pH of the electrolyte was adjusted by the addition of sodium hydroxide. It was seen that the pore diameter depended on the anodization voltage, whereas the nanotube length depended on both the electrolyte pH and anodization voltage. Nanotube lengths varied from 380 nm to 6 μm and pore diameters from 30 to 110 nm as the electrolyte pH (1.11–5) and the anodization potential (10–25 V) was changed. The as-prepared amorphous samples were crystallized by annealing at temperatures ranging from 370 to 630 °C in oxygen for 6 h.

Hu *et al.* [14] also synthesized TiO_2_ nanotube arrays by the anodization approach. A titanium foil was cleaned by soap, acetone, and isopropanol and used as an anode, whereas platinum foil was used as a cathode. The titanium foil was immersed in an electrolyte solution containing NH_4_F and dimethyl sulphoxide at a 45 V constant potential for 9 h. The obtained amorphous TiO_2_ nanotube arrays were annealed at 400 °C for 1.5 h. The resulting nanotubes were 350 nm in diameter and 3.5 μm in length with a wall thickness of 10 nm. The branched TiO_2_ nanotubes were obtained through a modification process on TiO_2_ nanotubes array by hydrothermal methods [14]. The as prepared TiO_2_ nanotube arrays were immersed in a solution containing HCl with constant stirring at 25 °C for 15 min. Titanium (IV) isopropoxide was dropped into the solution under constant stirring for 1 h, and then the beaker was sealed and heated at 95 °C for 9 h with slight stirring. After the reaction, the reactant was cooled to room temperature and washed with ethanol and distilled water. The as prepared branched TiO_2_ nanotube arrays were annealed in a muffle furnace at 400 °C for 2 h. It was observed that TiO_2_ nanocrystal nucleus formed on the rough surfaces of the TiO_2_ nanotubes with special bamboo structures with a larger and rougher surface area. Similarly, P25 (A commercial photocatalyst from Degussa, Germany) coated TiO_2_ nanotube arrays were synthesized by the hydrothermal approach on the uncoated TiO_2_ nanotube arrays [14]. In this process P25 was added to distilled water and then mixed vigorously by magnetic stirring and ultrasonicating followed by transferring into a Teflon-lined autoclave. The autoclave was sealed and heated to 80–120 °C for 12 h to coat P25 on the TiO_2_ nanotube arrays, and then it was cooled to room temperature and washed with distilled water. The P25 coated TiO_2_ nanotube arrays were annealed at 400 °C for 2 h.

A novel approach was developed for the production of nanofibers of pure TiO_2_ and mixed oxide of TiO_2_ and SnO_2_ through a nanocarving process [25,80]. Pure TiO_2_ nanofibers were produced by sintering TiO_2_ nanoparticle (32 nm) pellets formed at 392 MPa in a temperature range from 1,100–1,400 °C [80]. The sintered TiO_2_ was shaped as a disk with a thickness of 1 mm and diameter of 10 mm that was exposed to an atmosphere of 5% H_2_ with balance N_2_ at 700 °C. Two types of gas flow rates (100 and 500 mL/min) were studied. It was found that samples sintered at 1,200 °C with a gas flow rate of 500 mL/min showed well developed fiber with a diameter of 15–50 nm and length of 1–5 μm. Similarly, for mixed oxide nanofibers, 90 mol% TiO_2_ and 10 mol% SnO_2_ powder was mixed in isopropanol followed by milling with yttria stabilized zirconia balls for 4 h [25]. After ball milling, the isopropanol was evaporated and the resulting powders were compacted into 12.7 mm disks at a peak stress of 392 MPa. The compacted disks were then sintered at two different temperatures, 1,450 °C and 1,200 °C for 2 and 6 h respectively. To create nanofibers, the sintered disks were exposed to 5% H_2_ in background N_2_ at 700 °C for 8 h under approximately 1,000 mL/min flow of gas. From XRD results it was seen that the TiO_2_–SnO_2_ mixture sintered at 1,450 °C represented only rutile and SnO_2_ was completely dissolved (solid solution) into the TiO_2_ matrix. On the other hand, SnO_2_ peaks were found for the TiO_2_–SnO_2_ samples sintered at 1,200 °C, which indicated that the mixture went through spinodal decomposition. During the nanocarving process for 2 h in the solid solution sample nanofibers were not evident. However, for the 6 h solid solution samples, nanofibers were obvious and grains become faceted. From this, the authors claimed that faceted grains with rutile structure were beneficial for nanofiber formation. The spinodally decomposed samples nanocraved for 6 h had well defined and oriented fibers with grooves on the grains which ensured enhanced surface area.

Francioso *et al.* [26] developed a nanofabrication process for the production of polycrystalline TiO_2_ nanowire arrays by 365 nm UV lithography and dry plasma etching on silica substrate. A thin layer of TiO_2_ was deposited on the silica substrate by sol-gel methods. Calcination was carried out at 500 °C to obtain the anatase phase of TiO_2_ film. A thin layer of photoresist was spun onto the film surface with array structures of 500 nm width and 800 nm of pitch. High pressure plasma was adopted in an Oxford Plasmalab 80 RIE reactor to perform micromachining of TiO_2_ thin films at 200 mTorr and SF_6_ chemistry. The etching time was about 390 s. The resulting nanowire arrays were 90–180 nm in width and 1,400 μm in length.

Among the processing routes for the production of TiO_2_ nanostructures, the hydrothermal and anodization approaches are most commonly employed. Depending on the starting materials and process conditions, the crystal structure of TiO_2_ nanostructures varied from anatase, rutile, brookite and tolepidocrocite. It was also seen that the morphology of the nanostructures can be altered by combining two processes. As an example, branched nanotubes can be obtained by the combination of anodization and hydrothermal processes. It is also seen that the anodization voltage has an effect on the pore diameter and pH has effect on the length and diameter of the nanostructure. Generally, with an increase in the anodization voltage and pH, the diameter and length of the nanostructure also increase. The as-grown nanostructures produced by anodization are mostly nanotube arrays with an amorphous crystal structure. However, annealing can be performed (>400 °C) to crystallize the nanostructure to either anatase or rutile. The H_2_-etching of TiO_2_ (nanocarving process) is a novel approach and provides an avenue for gas-phase assisted nano-machining of ceramics.

#### 4.3.2. Sensing Performance of TiO_2_ 1-D Nanostructures

Varghese *et al.* [44] grew TiO_2_ nanotubes arrays on Ti foil by an anodization process. The TiO_2_ nanotubes exhibited anatase phase at the nanotube walls and rutile phase at the barrier layer. They were able to detect H_2_ at temperatures as low as 180 °C. TiO_2_ nanotubes with a smaller pore diameter (46 nm) had higher sensitivity compared to larger pore diameters (76 nm) towards H_2_ gas. Generally, the sensitivity of TiO_2_ nanotubes increased with increasing temperature showing a variation of three orders in magnitude of resistance to 1,000 ppm of H_2_ at 400 °C. Conversely, the response time decreased with increasing temperature. It was seen that at 290 °C the response time was approximately 3 min. The sensors showed high selectivity to H_2_ compared to CO, CO_2_ and NH_3_. The high sensitivity of the nanotubes was due to H_2_ chemisorption onto the TiO_2_ surface where they acted as electron donors. TiO_2_ nanotube arrays (pore diameter 30 nm, wall thickness 13 nm, length ∼1 μm) having a crystalline structure showed the highest resistance variation, 8.7 orders of magnitude for 1,000 ppm H_2_ [79]. The ultra high response of this sensor is believed to be due to the highly active surface states on the nanotube walls, high surface area of the nanotube architecture, and the ordered geometry of the tube to tube electrical connections. Rout *et al.* [59] synthesized TiO_2_ nanowires with rutile structure for the detection of H_2_ gas at room temperature in presence of dry air. It was seen that at room temperature TiO_2_ nanowire showed sensitivity of 8 at 1,000 ppm H_2_ concentration.

It was seen from the work of Han *et al.* [75] that Pt and Pd nanoparticles on TiO_2_ nanotubes had a response almost twice that of TiO_2_ nanoparticles or nanotubes. Noble metals, such as Pt or Pd, activate the oxidation reaction because the heat of adsorption of oxygen on noble metals is sufficiently low. This phenomenon creates relatively low activation energy for oxidation and consequently a rapid rate of reaction. It was seen that the optimum temperature for maximum response of Pt and Pd nanoparticles-TiO_2_ sensor was around 250 °C. It was claimed that at this temperature the rate of reaction on the catalytic surface is the fastest, resulting a large change of voltage in the circuit. This also suggests that the higher response of Pt and Pd nanoparticle-TiO_2_ is due to the higher number of adsorption sites or the catalytic surface area. This is only possible if the size of Pd or Pt particles on TiO_2_ nanotubes is in the nano-scale, which was revealed from the TEM images of Pt and Pd nanoparticles on TiO_2_ nanotubes in Figure 8(a). Another possible reason for the enhanced response of Pt and Pd nanoparticle-TiO_2_ nanotubes is due to increased adsorption of hydrogen on the TiO_2_ nanotube surface which facilitates the hydrogen oxidation reaction by the Pd and Pt catalysts.

Development of mixed oxide nanostructure is another approach to investigate the performance of a TiO_2_-based sensor. Carney *et al.* [25] synthesized Ti_0.9_Si_0.1_O_2_ nanofibers by the nanocarving process. Due to the difference in the sintering temperature both solid solution (1,450 °C) and spinodally decomposed (1,200 °C) of Ti_0.9_Si_0.1_O_2_ samples were obtained which upon H_2_-etching produced nanofiber and nano-lamelar structure, respectively. Both these samples showed good sensitivity toward H_2_ gas with a response of ∼1.3 for 2% H_2_ at 400 °C. The response time and recovery time was 1–2 min and 5–7 min, respectively.

Comparing the sensitivity of anatase and rutile nanostructures it is seen that anatase polymorph of TiO_2_ has high sensitivity towards reducing gases like H_2_ and CO [81–83]. As a probable reason, it was claimed that the diffusing hydrogen atoms go to the interstitial sites [83,84] and as the c/a ratio of anatase is almost four times that of rutile, anatase lattice accommodates hydrogen more easily and hence has a higher sensitivity to hydrogen.

The sensitivity of nanowire arrays on silica fabricated by Francioso *et al.* [26] was studied for ethanol sensing. It was seen that the sensitivity of the sensor was approximately 50 at 550 °C for 2% and 3% ethanol concentrations. Comparing these results to the response of TiO_2_ thin film, the nanowire array showed higher sensitivity towards ethanol. The response is less than 10 in the case of TiO_2_ thin film for 2 and 3% ethanol concentrations at 550 °C. Hu *et al.* [38] synthesized four types of TiO_2_ nanobelts (TiO_2_ untreated nanobelts, TiO_2_ surface-coarsened nanobelts, Ag nanoparticles-TiO_2_ untreated nanobelts and Ag nanoparticles-TiO_2_ surface-coarsened nanobelts) for the detection of ethanol vapor. It was seen that Ag nanoparticles-TiO_2_ surface-coarsened nanobelts exhibited the best performance in ethanol vapor detection. The response was 46–153 at 200 °C for 500 ppm ethanol. The optimum working temperature was in the range of 200–250 °C. The response and recovery times were only 1–2 s for ethanol vapor detection.

Biao *et al.* [50] compared the sensitivity of Cu-doped and undoped TiO_2_ nanofibers for CO detection. It was observed that Cu-doped TiO_2_ nanofibers showed much higher sensitivity compared to pure TiO_2_ nanofibers. The sensitivity of Cu-doped TiO_2_ nanofibers was approximately 21, 17 times larger than pure TiO_2_ at 300 °C for 100 ppm CO. The maximum sensitivity of Cu-doped TiO_2_ was attained at 300 °C with a response and recovery time of 4 and 8 s, respectively. It was also seen that the Cu-doped TiO_2_ was less sensitive to CH_4_, CH_3_OH, C_2_H_5_OH, C_2_H_2_, H_2_ and NO.

Landau *et al.* [76] measured the sensitivity of TiO_2_ nanofibers towards NO_2_ gas. The response was measured in terms of I/I_o_ which was equivalent to R_o_/R. It was seen that the sensitivity decreased with increase in temperature from 300 °C to 400 °C. For example, the sensitivity to NO_2_ 250 ppb was found to be 74.3 at 300 °C and 3.3 at 400 °C. On the other hand, the response time increased with increase temperature and decreased with increase in concentration of NO_2_ gas.

An amorphous TiO_2_ nanotube array was synthesized by the anodization process for the detection of O_2_ [78]. It was seen that the sensitivity of amorphous TiO_2_ nanotube arrays roughly increased with increasing temperature, but above 180 °C exhibited irregular fluctuations with a very poor recovery. However, at 100 °C, high sensitivity, excellent recovery and a linear relationship with oxygen concentration was observed. At 100 °C, the amorphous TiO_2_ nanotube arrays exhibited sharp change in electrical resistance up to two orders of magnitude with change in O_2_ concentration. Comparing with other metal-oxide sensors such as Ga_2_O_3_ thin film (∼1.5) [85], nanoscale TiO_2_ thick film (∼1.5) [86] and SrTiO_3_ thick film (∼6.5) [87], amorphous TiO_2_ nanotube array showed higher response towards oxygen at 100 °C.

Generally, TiO_2_ is annealed in air or oxygen atmosphere at an elevated temperature to form a crystalline structure for the sensing of H_2_, CO, NO_2_ and CH_4_ [88–91]. The transformation of amorphous TiO_2_ anatase and rutile occurs during the annealing process. Crystalline TiO_2_ is highly advantageous for H_2_ detection but for oxygen, crystalline TiO_2_ exhibits a very poor recovery [44,78]. Gas sensing response for 1-D nano-structured TiO_2_ is summarized in Table 7.

### 4.4. 1-D In_2_O_3_ Nanostructured Gas Sensors

#### 4.4.1. Growth and Characterization of In_2_O_3_ Nanostructures

The processing techniques used to produce In_2_O_3_ nanostructures for gas sensing can be categorized as wet processing, solid-state processing, vapor-phase processing and hybrid processing. The wet processing routes include both electrospinning and sol-gel processes. Carbothermal reduction is the only solid-state processing route reported for the production of In_2_O_3_ nanowires. Chemical vapor deposition is one of the most employed vapor-phase processing routes for the synthesis of In_2_O_3_ nanostructures. Solvothermal is a hybrid processing route which consists of wet (hydrothermal) and solid-state (calcination) processes. Depending on the processing routes and experimental conditions, different surface morphologies with varying dimensions of In_2_O_3_ nanostructures were obtained. The processing details for the production of 1-D In_2_O_3_ nanostructures are summarized in Table 8.

**Figure 9.**
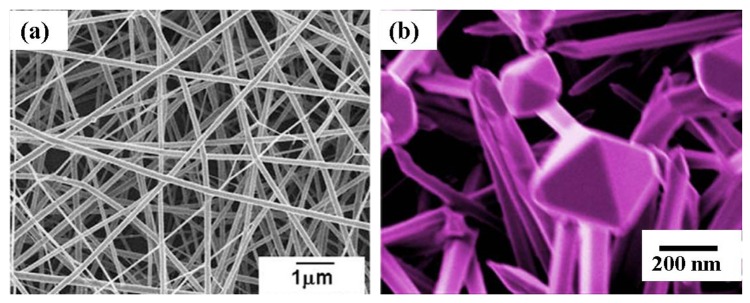
Morphology of In_2_O_3_: (**a**) SEM micrograph of nanofibers grown by electrospinning [93]. (**b**) Field emission scanning election microcopy (FESEM) micrographs of nanopushpins grown by chemical vapor deposition [29].

Zheng *et al.* [92] synthesized In_2_O_3_ nanofibers by electrospinning for C_2_H_5_OH gas sensing. In this procedure, In(NO_3_)_3_-4.5H_2_O powder was added to a mixed solvent of *N,N*-dimethylformamide and ethanol in the weight ratio of 1:1. This solution was stirred vigorously for 2 h. After that PVP was added to the above solution and stirred for 6 h. This solution was loaded into a plastic syringe and connected to a DC voltage supply of 15 kV. An aluminum foil served as the counter electrode. Distance between the capillary and the electrode was 20 cm. The as-spun PVP/In(NO_3_)_3_-4.5H_2_O composite nanofibers were placed in a vacuum oven for 12 h at room temperature in order to remove the residual solvent, and then calcined in air from 500–800 °C for 4 h. From the XRD results it was seen that the electrospun PVP/In(NO_3_)_3_-4.5H_2_O nanofibers were amorphous and after calcinations, the In_2_O_3_ exhibited a cubic structure. With increasing in temperature the crystallinity in In_2_O_3_ also increased. After calcination the final diameter of In_2_O_3_ nanofibers were 60–100 nm with lengths up to several tens of micrometers. The sensitivity of the In_2_O_3_-based sensors was altered by depositing different nanoparticles on the nanostructure surface. As an example, Pt nanoparticles were deposited on electrospun In_2_O_3_ nanofibers for H_2_S detection [93]. To load Pt nanoparticles on In_2_O_3_ nanofibers, the as-prepared nanofibers (Figure 9(a)) and H_2_PtCl_6_ aqueous solution were added into water and heated to boiling for 30 min [92]. Then, sodium citrate aqueous solution was added rapidly and the mixture was kept at a boiling temperature for 30 min. The Pt nanoparticles on In_2_O_3_ nanofibers could be separated through centrifugation and washed with deionized water for several times. Then the sample was dried at 60 °C. It was observed that Pt nanoparticles of 5–10 nm in size were randomly distributed on the surface of the In_2_O_3_ nanofibers.

The sol-gel technique has been employed for the synthesis of In_2_O_3_ nanorods for H_2_ gas detection [15]. In a typical experiment, InCl_3_-4H_2_O and sodium dodecyl sulfate were dissolved in water and stirred at 60 °C for 20 min. Sodium hydroxide solution was added to the above solution under continuous stirring at 60 °C until a pH of 12 was obtained. After aging at room temperature for 12 h, the precipitate was centrifugally separated, washed with deionized water and dried in air at 60 °C for 12 h. The as obtained nanorods had diameters of 70–100 nm with lengths of 300–900 nm. The side view of the sample exhibited the surface of the rods to be rough. High-resolution transmission electron microscopy (HRTEM) of the as prepared In_2_O_3_ nanorods possessed a porous structure with pore sizes in the range of 5–10 nm.

Carbothermal reduction is another method for the production of In_2_O_3_ nanowires. A mixture of ground In_2_O_3_ and active carbon was taken in an alumina boat and placed inside a horizontal tube furnace [51]. Then, under constant flow of N_2_, the furnace was heated to 1,000 °C and held at this temperature for 180 min. After the furnace cooled to room temperature, the In_2_O_3_ nanowires were found on the wall of alumina boat. From TEM image analysis it was seen that the nanowires had a diameter of 60–160 nm and length of 0.5 to a few micrometers.

Nanowires and nanoneedles were grown on a silicon substrate by chemical vapor deposition process for H_2_ gas sensing [29]. In this procedure, high purity indium grains were placed on an alumina boat inside a quartz tube. A silicon wafer coated with 10 nm Au layer was placed above the indium grains. The tube was heated to a target temperature for 1 h and Ar gas was flown at a rate of 100 mL/min. In order to study the effect of temperature on the morphology of In_2_O_3_ nanostructures, the synthesis was carried out over the range 700 to 900 °C. It was seen that nanorods formed at the synthesis temperature of 700 °C while nanowires and nanoneedles formed at 800 and 900 °C, respectively. It was also seen that the diameter and length of nanowires (diameter: 70–80 nm, length: several micrometers) were somewhat smaller than nanoneedles (diameter: 150–200 nm, length: 4–5 μm). The XRD results indicated that the nanowire had the cubic phase of In_2_O_3_. It was found that Au played an important role for the production of nanowires. It was seen by Qurashi *et al.* [29] that the nanowires were terminated in their growing ends by Au nanoparticles. The presence of Au nanoparticles at the end of the nanowires indicated the vapor-liquid-solid growth mechanism. No metal drops were observed in the case of nanoneedles, which indicated a vapor-solid mechanism.

Nanopushpins of In_2_O_3_ were also obtained on a silicon wafer substrate by chemical vapor deposition as it is seen in Figure 9(b) [30]. High purity indium particles were placed at one end of an alumina boat and kept inside a quartz tube. A silicon wafer was placed at the center of the boat and subsequently heated up to 800 °C for 1 h with a constant flow (100 mL/min) of 98% Ar and 2% O_2_. After this In_2_O_3_ nanopushpins were found deposited on silicon wafer. At high magnification it was found that each nanopushpin consisted of a nanorod stem with a tetrahedral tip. The nanorods had diameters of 80–120 nm with lengths of 500 nm to 1 μm.

Au nanoparticles were also deposited on the In_2_O_3_ nanostructures to increase the sensitivity of the sensor. One such technique was presented by Singh *et al.* [95] where In_2_O_3_ nanowires were grown in a horizontal chemical vapor deposition furnace at 900 °C in the presence of In_2_O_3_ with graphite powder and Ar gas. The flow rate of the Ar gas was fixed at 50 sccm with 1 mbar pressure. A silicon wafer with a 9 nm Au layer was kept downstream as a substrate. The substrate temperature ranged from 400 to 550 °C for duration of 60 min of deposition. For the preparation of Au nanoparticles, HAuCl_4_-3H_2_O and sodium citrate was dissolved in deionized water followed by refluxing at 115 °C and cooling to room temperature.

The previously prepared In_2_O_3_ nanowires deposited on Si substrate were treated with a mixture of hydrogen peroxide, NH_4_OH and deionized water at 75 °C for 45 min. The substrate was then rinsed with deionized water, blown with nitrogen and dried at 100 °C under a vacuum for 30 min to create a surface rich in hydroxyl groups on the In_2_O_3_ nanowires surface to facilitate the silanization process. The hydroxyl terminated substrates were rinsed with toluene and then immersed in a 3 mM p-aminophenyltrimethoxysilane solution in toluene for 2 h. Subsequently, the substrate was removed from the solution, rinsed with toluene followed by acetone and finally blown dry with nitrogen. The silane treated Si substrate was immersed in the freshly prepared Au nanoparticles solution for 60 min, rinsed with deionized water, and then baked at 110 °C for 5 min to remove residual moisture. In this process the *p*-aminophenyltrimethoxysilane layer was used to functionalize the Au nanoparticles on the nanowire surface. The nanowires showed a high coverage of Au nanoparticles (∼10 nm) on the surface as seen in by TEM.

Similarly, Pt nanoparticles were also deposited on the In_2_O_3_ nanowires grown by chemical vapor deposition [24]. In this procedure, indium powders and Mg nanopowders were mixed in a weight ratio of 1:1 on a silicon substrate having 3 nm Au layer placed inside a quartz tube in a vertical furnace. A mixture of 97% Ar and 3% O_2_ gas was flown at rate of 2 L/min at 800 °C. Subsequently, the substrates were transferred to a turbo sputter coater. Sputter deposition was conducted by using a Pt target in high purity Ar ambient for 40 s at room temperature. A DC current of 10 mA was maintained during sputtering. The as prepared In_2_O_3_ core/Pt shell nanowires were annealed at 800 °C for 30 min in Ar ambient. The as synthesized In_2_O_3_ core/Pt shell nanowire composed of a rod like In_2_O_3_ core and Pt shell with an approximate thickness of 2 nm.

Solvothermal is another process that has been employed for the growth of In_2_O_3_ nanorods. Single-crystal, metastable, hexagonal In_2_O_3_ nanorods were synthesized through the annealing of InOOH nanorods by solvothermal method under ambient pressure [94]. In this procedure the reaction medium was prepared by using oleic acid, n-amyl alcohol, and n-hexane. In(NO_3_)_3_ and NaOH solutions were mixed in a volume ratio of 1:1 and added to the previous solution with vigorous stirring. The obtained emulsion was taken to an autoclave and heated at 200 °C for 20 h followed by cooling to room temperature. The precipitate was washed by absolute ethanol and distilled water and dried at 60 °C for several hours. The resulting InOOH nanorods were calcined at 600 °C for 1 h to produce In_2_O_3_ nanorods. From TEM images it was revealed that the diameter of the nanorods were 20–50 nm with a length of more than 100 nm.

From the above literature survey it can be seen that different morphologies of In_2_O_3_ nanostructures can be produced depending on the processing route. The nanostructures produced can be varied to include nanorods, nanotubes, nanowires, nanofibers, nanoneedles and nanopushpins. Furthermore, the nanostructure's surface can be modified by depositing Pt and Au nanoparticles to achieve a better sensitivity. In the electrospinning process, the obtained nanostructure exhibits an amorphous structure due to the low process temperature. However, the crystallinity of the nanostructure can be increased by calcination in the temperature range of 500–800 °C. The temperature in the chemical vapor deposition process has effect on the In_2_O_3_ nanostructure morphology. It was found by Qurashi *et al.* [29] that at 700, 800 and 900 °C the morphology produced was nanorod, nanowire and nanoneedle, respectively. Also, an Au layer on silicon substrate used in the chemical vapor deposition process had a direct effect on the nanowire growth process. Existence of Au at the nanowire tip suggested that the vapor-liquid-solid growth process was involved [24,29].

#### 4.4.2. Sensing Performance of In_2_O_3_ 1-D Nanostructures

The sensing characteristics of In_2_O_3_ nanostructures were examined for H_2_, H_2_S, ethanol, CO and O_2_ gases of different concentrations. The morphology of the nanostructures was varied form nanorods, nanowires, nanofibers, nanoneedles to nanopushpins. Additionally, the surface of the In_2_O_3_ nanostructures could be functionalized with different nanoparticles such as Pt and Au. These kinds of morphological enhancements showed increased sensitivity to different gases with a varying degree of success. The summarized results based on sensitivity of 1-D In_2_O_3_ nanostructures are presented in Table 9.

The sensitivity of In_2_O_3_ nanowires and nanoneedles towards H_2_ gas was measured by Qurashi *et al.* [29] at 200 °C. It was seen that the resistance of the sensor decreased as the H_2_ concentration increased from 500 to 1,500 ppm. In general, nanowires exhibited higher response compared with the nanoneedles and it was believed that the higher response was associated with the high surface to volume ratio of the nanowires. The response time decreased with increase in temperature. For In_2_O_3_ nanowires the response and recovery time was 31 s and 80 s, respectively at 200 °C for 500 ppm H_2_ concentration. On the other hand, the response time of the nanoneedle was 60 s. Similarly, the sensitivity of In_2_O_3_ nanopushpins towards H_2_ was evaluated and a dynamic and swift response was found at 250 °C [30]. In this case, it was also seen that as the concentration of H_2_ gas and temperature were increased the resistance of the sensor decreased. The response time and recovery time for 500 ppm H_2_ were near 35 s and 60 s, respectively. Porous In_2_O_3_ nanorods showed optimum detection of H_2_ at 340 °C with a response of ∼ 6 to 500 ppm of H_2_ [15].

Pt nanoparticles on In_2_O_3_ nanofibers improved the response of the sensor towards H_2_S gas [93]. 2.3 wt% Pt loaded In_2_O_3_ nanofibers exhibited a response of 1,490 at 600 ppm H_2_S at 200 °C compared to 34 and 150, respectively for In_2_O_3_ film and pure In_2_O_3_ nanofibers. The response was higher in the In_2_O_3_ nanofibers compared to In_2_O_3_ film due to high surface to volume ratio of the nanofibers. Additionally, the higher response in the Pt nanoparticles loaded In_2_O_3_ nanofibers is due to enhanced catalytic adsorption of gas molecules that accelerates the electron exchange between the sensor and H_2_S gas. Due to the catalytic activation upon the addition of Pt nanoparticles, the optimum working temperature is lower in the Pt nanoparticle added In_2_O_3_ sensor (200 °C) compared with the pure In_2_O_3_ nanofibers (260 °C). The response and recovery times of the Pt nanoparticles loaded In_2_O_3_ sensor were 60 s and 120 s, respectively.

Zheng *et al.* [92] utilized In_2_O_3_ nanofibers for sensing of C_2_H_5_OH gas. It was seen that the response of the sensor increased sharply as the concentration of C_2_H_5_OH was raised from 100 to 5,000 ppm. As the C_2_H_5_OH concentration exceeded 5,000 ppm, the response changed slowly and gradually reached saturation. In_2_O_3_ nanofibers showed a response of about 379 at 300 °C for 15,000 ppm C_2_H_5_OH concentration. The calcination temperature of the nanofibers also has an effect on C_2_H_5_OH gas sensing. As the calcination temperature was increased from 500 to 800 °C, the crystal structure of In_2_O_3_ changed from non-crystalline to crystalline. However, samples calcined at 700 °C showed the highest response towards C_2_H_5_OH gas and it was claimed that samples calcined below 700 °C might not possess sufficient crystallinity whereas, above 700 °C there could be grain growth and agglomeration which resulted in a decrease in surface area. The optimum working temperature was evaluated to be 300 °C with a response and recovery time of 1 s and 5 s, respectively. In_2_O_3_ nanowires produced by carbothermal reduction were also exploited for ethanol detection [51]. The response of In_2_O_3_ sensors towards ethanol gas were measured as a function of operating temperature. It was found that In_2_O_3_ sensors showed maximum response towards ethanol at 370 °C. For 1,000 ppm of ethanol, the maximum response was 25.3 at 370 °C. The sensor showed almost no response to 1,000 ppm CH_4_ and CH_3_OH gas when operated at 150–350 °C. The sensor exhibited lower response to 1,000 ppm (C_2_H_5_)_3_N and CH_3_COCH_3_ when operated in the range of 150–400 °C. This suggests that the sensor based on In_2_O_3_ nanowires is selective to C_2_H_5_OH gas at 370 °C. The response times and the recovery times were very short, about 10 s and 20 s, respectively. In_2_O_3_ nanorods produced by solvothermal method had a response of 11.5 to 50 ppm of ethanol at 330 °C [94]. Even for concentrations as low as 5 ppm, the sensitivity of In_2_O_3_ nanorod sensors could reach 1.84. The response and recovery properties of this sensor were quite short, 6 s and 11 s respectively. In addition, the reversibility and repeatability of these sensors were also very good. They were still sensitive to small concentration of ethanol (5 ppm) even after exposure in high concentration ethanol (1,000 ppm). Furthermore, the sensors were totally insensitive to CO and H_2_.

Au nanoparticles were loaded on In_2_O_3_ nanowires for the detection of CO gas [95]. Due to Schottky contact between the nanowire-electrode junctions a higher number of electrons were transferred to the nanowire channels during CO oxidation. It was seen that the response increased with increasing the Au nanoparticles loading. Nanowires with high coverage of Au nanoparticles at the surface showed a response of nearly 104 toward 5 ppm CO gas at room temperature. The response increased to as high as 23 times in the highly covered In_2_O_3_ nanowires compared with lightly covered by Au nanoparticles.

The response and recovery time for the Au nanoparticle functionalized In_2_O_3_ nanowire were found to be 130 s and 50 s respectively. Kim *et al.* [24] synthesized Pt-functionalized In_2_O_3_ nanowire sensor for oxygen sensing at 50 °C. The concentration of oxygen was varied from 10 to 400 ppm. Unlike reducing gases such as H_2_, H_2_S, and CO, the resistance of the sensors increased sharply after exposure to oxygen. When the oxygen supply was discontinued, the resistance quickly dropped to a low value. It was found that at 50 °C bare In_2_O_3_ nanowire sensors exhibited no sensitivity towards oxygen, whereas Pt functionalized nanowires were sensitive to oxygen.

In summary, the sensitivity of In_2_O_3_ nanostructures was examined for H_2_, H_2_S, ethanol, CO and O_2_ gas. The resistance of the sensors decreased when exposed to the reducing gases such as H_2_, H_2_S, CO and ethanol and increased when exposed to oxygen [24]. However, it was also seen that the sensitivity depended on the nanostructure morphology and crystal structure. Nanowires were found to have better response towards H_2_ gas compared to nanoneedles because of increased surface area [29]. Additionally, non-crystalline nanostructures had lower response compared with crystalline nanostructures. It was also claimed that nanostructures calcined at higher temperature had a larger grain size which resulted in a lower response [92]. Nanostructures loaded with nanoparticles showed very high sensitivity compared with the unloaded nanostructures. One such example was found for H_2_S sensing where the sensitivity increased from 150 to 1,490 with the loading of 2.3 wt% Pt nanoparticles [93]. It was suggested that nanoparticles enhanced the catalytic adsorption of gas molecules and accelerated the electron exchange rate which in turn showed better sensitivity.

### 4.5. Non-Conventional 1-D Nanostructured Gas Sensors (WO_x_, AgVO_3_, CdO, MoO_3_, CuO, TeO_2_, and Fe_2_O_3_)

#### 4.5.1. Growth and Characterization of Non-Conventional Nanostructures

Different types of non-conventional 1-D metal-oxide nanostructures, such as WO_x_, AgVO_3_, CdO, MoO_3_, CuO, TeO_2_ and Fe_2_O_3_ were synthesized and investigated for different gases. Due to lack of sufficient data, the results are discrete though some are promising for sensing applications. Table 10 summarizes the processing parameters and morphologies of different non-conventional metal-oxides.

Tungsten oxide (WO_2.72_) nanowires were prepared by the solvothermal synthesis for H_2_ and LPG sensing [59,96]. In this procedure, 1 gm of tungsten hexachloride was placed in an autoclave filled with ethanol up to 90% of its volume. The synthesis was carried out at 200 °C for 24 h. The product obtained by centrifugation was washed with ethanol. From TEM analysis it was seen that WO_2.72_ nanowires were monoclinic with a diameter of 5–30 nm and length of 100–500 nm. Hieu *et al.* [97] studied the growth of WO_3_ nanowires on porous single wall carbon nanotubes (SWCNTs) by thermal oxidation process. In this process, SWCNTs were grown on SiO_2_/Si substrate in an arc-discharge chamber [98]. A tungsten layer (100 nm) was deposited on the SWCNTs by DC sputtering. Finally, the nanowire coated SWCNTs were oxidized at 700 °C in a tube furnace for 2 h. It was found that this temperature was high enough to burn out the SWCNTs [98]. From FESEM images it was seen that the tungsten nanowires appeared as an agglomeration of nanoparticles rather than a continuous tube shape. WO_3_ nanowires were monoclinic in structure with a diameter of 70 nm and a length of several micrometers.

β-AgVO_3_ nanowires were successfully prepared by ultrasonic treatment followed by a hydrothermal reaction using V_2_O_5_ sol [34,35]. The as-prepared V_2_O_5_ sol and Ag_2_O powder were mixed by stirring and ultrasonication. Then the mixture was transferred into a Telfon-lined stainless steel autoclave and kept at 180 °C for 1 day. The products were collected and washed repeatedly with distilled water and finally dried at 80 °C in air for 12 h. The resulting nanowires had 50–100 nm thickness and 100–700 nm width. The XRD pattern of the as-synthesized product confirmed the monoclinic phase of β-AgVO_3_ nanowires.

Highly porous CdO nanowires were grown by Guo *et al.* [99] by a hydrothermal process for NO_2_ detection. In a typical synthesis, CdCl_2_·2.5H_2_O was dissolved into distilled water with magnetic stirring. Then ethylenediamine, Na_2_CO_3_ and NH_3_ solution were added followed by transferring into a Teflon-lined stainless steel autoclave at 180 °C for 24 h. The white flocculate precursor nanowires were isolated using centrifugation and washing with distilled water and ethanol. The as-synthesized precursor nanowires were dried in a vacuum oven at 50 °C for 4 h. The TEM and SEM results showed that the precursor had a diameter of 120 nm with a length of 100 μm. The XRD results showed the cubic structure of the precursor. The precursor was calcined in the temperature range of 300–650 °C. It was found that at 300 °C a porous structure began to form and after 600 °C the structure started to collapse. However, the optimum calcination temperature for obtaining a porous CdO nanowire was found to be 500–550 °C.

MoO_3_ needles were synthesized by sol-gel technique through molybdenum iso-propoxide [39]. Lamellar MoO_3_ was synthesized by thermal evaporation process [100]. MoO_3_ powder was placed at the centre of a furnace at 770 °C with a substrate 12 cm away from it. Thermal deposition was carried out using 10% O_2_ with balanced Ar. The resulting lamellar MoO_3_ had orthorhombic structure with a thickness of 500 nm and width of 5 μm.

Single crystalline CuO nanoribbons containing substantial amounts of nanorings and nanoloops were synthesized by a surfactant-assisted hydrothermal route [101]. Briefly, sodium dodecyl-benzenesulfonate was added to CuSO_4_ solution with continuous stirring followed by NaOH addition. The mixture was hydrothermally treated at 120 °C for 10 h and washed with distilled water and absolute ethanol followed by drying under a vacuum at 60 °C for 4 h. The resulting nanoribbons had a 2–8 nm thickness and 30–100 nm width. The nanoribbons were functionalized with Pt and Au through a wet-chemical reduction method. The as-prepared nanoribbons were ultrasonically dispersed in H_2_O with H_2_PtCl_6_ or HAuCl_4_ and L-ascorbic acid solution. The obtained mixture was heated at 60 °C for 15 min under continuous stirring.

TeO_2_ nanowires with a tetragonal structure were grown by Liu *et al.* [102] using thermal evaporation. High purity tellurium metal was put into an alumina crucible with a silicon wafer 2 mm above. The system was covered and heated in a muffle furnace at 400 °C for 2 h. Nanowires with a diameter of 30–200 nm were deposited on the lower surface of the Si wafer.

Porous urchin-like α-Fe_2_O_3_ nanostructures were prepared by Hao *et al.* [31] by hydrothermal treatment followed by a calcination processes. In a typical procedure, FeSO_4_·7H_2_O and CH_3_COONa·4H_2_O were dissolved in deionized water at room temperature. After stirring vigorously for a period of time at 60 °C, the yellow slurry was centrifuged and washed several times with distilled water and absolute alcohol and dried at 70 °C. The final products of porous α-Fe_2_O_3_ nanostructures were obtained by calcining the as-prepared α-FeOOH precursors at 500 °C for 3 h in air. The crystal structure of the nanostructures was examined by XRD and was found to be hexagonal in phase. It was seen by SEM that the samples had a uniform urchin shape with a diameter of 1 μm. The urchins consisted of several straight and porous nanorods radiating from the center. The average diameter of the nanorods was 30–40 nm with lengths of approximately 500 nm. Table 10 summarizes the processing parameters of oxide 1-D nano-strucres for non-conventional sensors.

#### 4.5.2. Sensing Performance of Non-Conventional 1-D Nanostructures

The sensing performance of 1-D metal-oxides of WO_x_, β-AgVO_3_, CdO, MoO_3_, CuO, TeO_2_, α-Fe_2_O_3_ were evaluated for different gases. Both reducing and oxidizing environments were studied. Because of limited number of studies, the results are not directly comparable, but show some interesting characteristics and are summarized in Table 11.

WO_2.72_ nanowires grown by solvothermal route showed good response towards H_2_ and LPG gas in presence of dry air [59]. It was seen that the resistance of WO_2.72_ nanowires decreased when exposed to these reducing gases. However, it was also seen that the sensitivity of nanowires having a 40 nm diameter was higher compared to nanowires having only a 16 nm diameter, which was unexpected. The response for 1,000 ppm H_2_ at 25 °C was 22. WO_2.72_ nanowires of 40 nm diameter also showed good response of approximately 15 toward 1,000 ppm LPG. The response and recovery times of the nanowires were 38 s and 28 s respectively. The WO_3_ nanowires showed a linear relationship for NH_3_ gas detection, *i.e.*, with increasing temperature the sensitivity increased linearly [97]. The optimum working temperature for NH_3_ detection was measured to be 250 °C. The sensitivity for 1,500 ppm NH_3_ was found to be 9.67 at 250 °C. The response and recovery time showed dependence on temperature and optimum results and were found to be 7 s and 8 s respectively at 250 °C. Mai *et al.* [35] developed β-AgVO_3_ nanowires through ultrasonic treatment followed by hydrothermal reaction for H_2_S detection. It was found that the sensitivity increased with increasing H_2_S concentration from 50 to 400 ppm. The sensor exhibited a linear relationship with a threshold switching of 6 V to switch the individual nanowire from nonconductive to conductive. The sensitivity was 1.12 for 400 ppm H_2_S at 250 °C. However, there was little sensitivity toward H_2_ or CO gas. The response and recovery times were less than 10 s and 20 s respectively.

The resistance of the CdO nanowires increased remarkably upon exposure to oxidizing gases such as NO_x_ [99]. With increasing NO_x_ concentration from 1 ppm to 300 ppm, the sensitivity increased and reached saturation at 150 ppm. The sensitivity measured for 150 ppm of NO_x_ was above 150.

MoO_3_ needles were prepared by Galatsis *et al.* [39] via a sol-gel technique. It was found that MoO_3_ exhibited a higher response towards O_2_ compared to WO_3_. The response to 1,000 ppm of O_2_ at 370 °C was 39 with a response and recovery time of 1s and 5 s respectively. Towards ozone (O_3_), no response was shown by MoO_3_ needles due to high resistance. MoO_3_ lamellar showed increased resistance for oxidizing NO_2_ gas [100]. The response was about 1.18 towards 10 ppm NO_2_ at 225 °C, which was determined to be the optimum working temperature.

CuO is a p-type semiconductor and hence the resistance of the sensor increased when exposed to reducing gases such as; HCHO and ethanol [101]. However, it was seen that the sensing properties of CuO nanoribbons were better than CuO powder or nanoplates. The sensitivity of CuO nanoribbons were further improved by loading Pt and Au as shown in Table 11.

**Figure 10.**
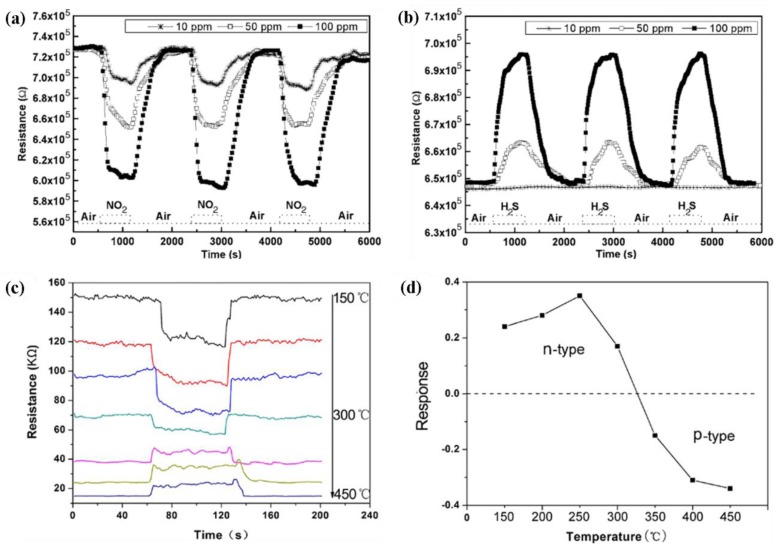
(**a**) Response of p-type TeO_2_ nanowires towards oxidizing NO_2_ gas [102]. (**b**) Response of p-type TeO_2_ nanowires towards reducing H_2_S gas [102]. (**c**) and (**d**) Dynamic response of α-Fe_2_O_3_ porous urchin toward 10 ppm H_2_S at different temperatures [31].

The sensitivity of TeO_2_ nanowires were measured in terms of resistivity by Liu *et al.* [102]. The resistance response of the TeO_2_ nanowires were measured from synthetic air to 10, 50, and 100 ppm NO_2_ gas at room temperature (26 °C). Since TeO_2_ is a p-type metal-oxide, the sensor's resistance decreased upon the introduction of oxidizing gas such as NO_2_ (Figure 10(a)) and the sensor resistance increased when exposed to reducing gas such as H_2_S (Figure 10(b)). The response time of the nanowires was about 2 min.

An interesting behavior was seen by n-type α-Fe_2_O_3_ porous urchin where the response behavior to H_2_S changed with an increase in temperature [31]. It can be seen from Figure 10(c,d) that for 10 ppm of H_2_S the response behavior changed from n-type to p-type with an increase in working temperatures. Below 300 °C, the sensor showed n-type response while at temperatures above 350 °C the response was p-type. These results clearly indicate a switching from n-type to p-type behavior with an increase in working temperature. Similar response behavior was observed in some other reducing gases including ethanol, methanol and acetone. However, the maximum n-type response for H_2_S was seen at 250 °C with a response and recovery time of 5 s and 10 s, respectively.

The sensing behaviour of non-conventional metal-oxides shows some interesting characteristics. The resistance change upon exposure towards reducing and oxidizing gas was utilized to measure the sensitivity. The 1-D nanostructures can be grouped into n-type (WOx, β-AgVO_3_, CdO, MoO_3_) and p-type (CoO, TeO_2_) categories. It is seen that, for n-type material the resistance is decreased when exposed to reducing gases and the opposite is observed for p-type materials as expected. However, α-Fe_2_O_3_ porous urchin showed n- to p-type transition with increasing temperature and gas concentration.

## 5. Sensing Mechanism of 1-D Nanostructured Gas Sensors

**Figure 11.**
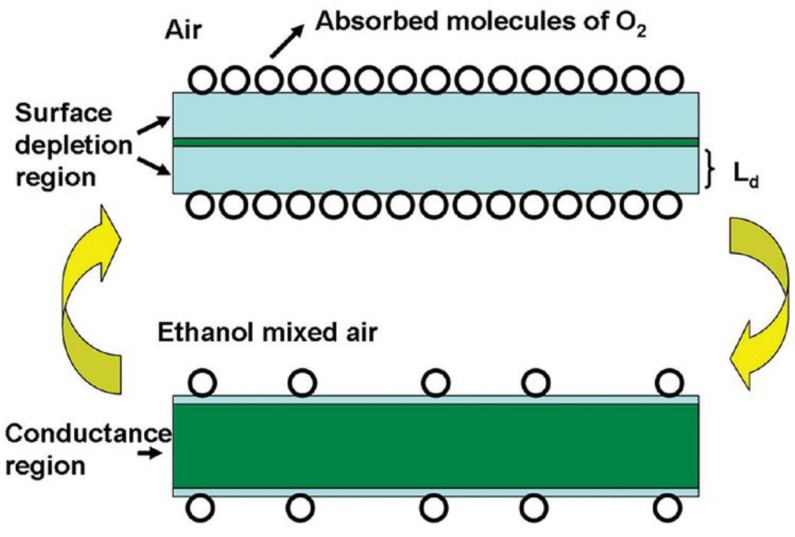
Sensing mechanism of TiO_2_ nanobelts to ethanol. Reprinted with permission from Ref. [38]. Copyright (2010) American Chemical Society.

It is well agreed that when 1-D metal-oxide nanostructures are exposed to air, oxygen molecules are adsorbed on the surface of the nanostructures and an O_2_^−^ ion is formed at the surface by capturing an electron from the conduction band. This results in a depleted of electrons at the nanostructure's surface and a high resistance in air ambient (Figure 11). The thickness of surface depletion layer might vary from semiconductor to semiconductor. For example, the surface depleted layer thickness is approximately 10–25 nm for TiO_2_ nanobelts [38], whereas, for ZnO it is several nanometers [103]. However, when n-type semiconductors (ZnO, SnO_2_, TiO_2_, In_2_O_3_, WOx, β-AgVO_3_, CdO, and MoO_3_) are exposed to a reducing environment (H_2_, H_2_S, HCHO, ethanol, *etc.*) at moderate temperatures, the gas reacts with the surface oxygen species and donates electrons. This results in a decrease in the resistivity of the nanostructures (Figure 11). This behavior is opposite when the n-type metal-oxides are exposed to an oxidizing gas such as NO_x_ [99]. However, for p-type semiconductors (example: CuO and TeO_2_), resistance is increased when exposed to reducing gases [101]. The major charge carriers are electrons and holes for n-type and p-type semiconductors, respectively.

In the case of film type sensors, the electrical modification only takes place in the grain boundary or porous surface [9]. On the other hand, for 1-D metal-oxide nanostructures this electrical modification takes place on the entire surface of the nanostructure [55,104]. It may be noted that 1-D nanostructures have high surface-to-volume ratio. The large depletion layer thickness combined with the high surface-to-volume ration results in a much larger change in conductivity for the nanostructures compared to thin films when exposed to an oxidizing or reducing. The morphology of 1-D nanostructures can be used to control the resistivity of the sensor which in turn controls the ultimate sensitivity of the device. For example, when nanorods having a vertically aligned [7], flower-like [57] or dendritic [20] nanostructure are exposed to air, the resistance of the sensor is increased due to presence of surface depletion layer in conjunction with the contact resistance of individual nanorods. This limits the electron transport between nanorods. Thereby, the total resistance of these types of nanostructures can be defined as the sum of bulk resistance (R_N_) and contact resistance (R_c_) as shown in the Equation (1) [7,57]:

(1) $$R=R_{c}+R_{N}$$

On the basis of this sensing mechanism, it can be seen that the sensitivity of the metal-oxide is strongly related to the charge transfer dynamics between the target gas molecules and the oxide matrix. One effective approach to improve the sensitivity of the metal-oxide is to deposit metal nanoparticles onto the metal-oxide surface. It was seen that the deposition of Pt nanoparticle on SnO_2_ [56] and In_2_O_3_ [93], Pd nanoparticles on SnO_2_ nanofibers [65], and Au nanoparticles on In_2_O_3_ nanowire [95] improved the sensitivity of the metal-oxide gas sensors by several times compare to those without the nanoparticles. Due to the presence of nanoparticles, the spillover effect is accelerated through “chemical sensitization” mechanism [105,106]. Specifically, nanoparticles can act as electron sinks because of large Helmholtz double-layer capacitance. When metal nanoparticles are deposited on a reducible oxide surfaces (e.g., ZnO, SnO_2_, TiO_2_, In_2_O_3_), partial charge transfer might occur from the center of oxide metal to the nanoparticles, leading to a negative charge accumulation on the nanoparticle surface. This could facilitate the dissociative adsorption of oxygen onto the particle surface and consequently enhance the formation of the electron depleted layer. Additionally, the deposition of metal nanoparticles onto the oxide surface, and hence the intimate interfacial contacts, may lead to the formation of structural defects which could serve as active surface sites for the adsorption of oxygen and target gas molecules.

It has also been found that Pd coating on ZnO nanorods improves the sensitivity by approximately 5 times for H_2_ gas detection compared to uncoated samples [23]. Catalytic dissociation of H_2_ to atomic hydrogen in presence of Pd was claimed as a possible reason for the increased sensitivity.

## 6. Summary and Future Directions

To date, one dimensional ZnO, SnO_2_, TiO_2_, In_2_O_3_, WO_3_, AgVO_3_, CdO, MoO_3_, CuO, TeO_2_, and Fe_2_O_3_ nanostructures have successfully been fabricated through different synthesis processes. A wide variety of surface morphologies can be achieved depending on the processing route employed. The synthesis processes for 1-D metal-oxide nanostructures can be grouped into wet processing, solid-state processing, molten-state processing, vapor-phase processing and hybrid processing. Some techniques have also been developed where the sensor device fabrication is integrated with the nanostructure growth [10]. For example, the on-chip fabrication of metal-oxide nanostructures on interdigitated substrate can be an attractive production route for commercial use. The procedure of on-chip fabrication of metal oxide is simpler compared with other sensor fabrication techniques like anodization, RF sputtering, molecular beam epitaxy, UV lithography, dry plasma etching *etc.* Thong *et al.* [10] grew SnO_2_ nanowires on Pt interdigitated Si substrate by thermal evaporation procedure. The dimension of the as-grown nanowires showed dependence on time and hence affect the performance of the sensor. This means that the dimension of the nanostructures is controllable during the production of sensor device which makes it flexible for fine tuning of the devise.

It was seen from the reported results that nanostructures having a rougher surface exhibited a higher response compared to those with smoother surfaces [5]. Additionally, nanostructures having vertically aligned [7], flower-like [53] and hierarchical dendritic [20] morphologies exhibited higher sensitivity due to the combination of bulk resistance and contact resistance. It was also seen that the surface of 1-D nanostructure modified by metal nanoparticles such as Pt on TiO_2_ nanotubes and In_2_O_3_ nanofibers [75,93], Pd on TiO_2_ nanotubes [75], and Au on In_2_O_3_ nanowires [95] showed very high sensitivity to different gases compared with their unmodified counterparts. Bulk property enhancement by doping such as Pt on SnO_2_ nanofibers [56], Pd on SnO_2_ nanofibers [65] and Cu on TiO_2_ nanofibers [50] showed improved and excellent sensing properties towards a variety of gases. For example, Pd coating on ZnO nanorods improved the sensor's response by a factor of five compared to uncoated nanorods through the catalytic dissociation of H_2_ [23]. Based on the reported results, a summary is given in Table 12 for the best choice of materials for sensing of a specific gas.

For example, vertically aligned ZnO nanorods show a sensitivity of 100 towards ethanol gas at 300 °C [7]. Though the sensitivity of flowerlike SnO_2_ nanorod is lower compared with the vertically aligned ZnO nanorods for ethanol sensing, the sensitivity reaches to 213 with the loading of L_a2_O_3_ on SnO_2_ nanorods [7,64]. Doping of Pt on SnO_2_ nanofibers showed the best result for ethanol sensing at 330 °C with a sensitivity of 1,020.6 [65]. Similarly loading of CuO nanoparticles on SnO_2_ nanoribbons showed the highest sensitivity for H_2_S sensing (18,000) at 50 °C [27]. Nanotube arrayed TiO_2_ showed the highest sensitivity (10^9^) towards H_2_ gas [79]. Fictionalization of In_2_O_3_ nanowires by Au nanoparticles has better sensitivity (104) at room temperature [95] compared with TiO_2_ nanofibers [50].

There are a few research gaps found in the 1-D nano-structure metal-oxide sensor field which are limiting further advancement. Very little work has been done on the lower limit of detection for a given gas. The optimum working temperature was not reported in all the studies. Also, the response and recovery times have not been reported in all cases. In some cases, the mechanism behind the formation of 1-D nanostructure and gas interactions on them are not well understood.

Future studies should be directed towards the formation of special structures specifically designed to enhancing sensing properties such as vertically aligned, flower-like, and hierarchical dendrites with the loading of nanoparticles and doping with different elements to improve sensor response. Special attention should be paid to the operating temperature for a given metal-oxide and sensor configuration such that a balance between sensing response and power consumption can be optimized. Developing systems that allow for the detection of very low gas concentrations at or only slightly above room temperature is of great importance. A lowered operating temperature results in less power consumption and a more energy efficient device. There is also a need for the development of sensors capable of operating in very harsh environments; both for industrial and safety industries. Through advancements in current capabilities to produce 1-D nanomaterials from a variety of semiconducting metal-oxides, techniques to modify and improve the bulk properties and surface configurations, and the fabrication of advanced sensor configurations the limitations and boundaries of chemical sensing are being ever expanded.

## Figures and Tables

**Table 1.** Fabrication parameters of tube-type gas sensors reported in literature.

**Sensor Materials**	**Sensor Material Morphology**	**Materials for Paste Formation**	**Ceramic Tube Material**	**Ceramic Tube Dimension**			**Electrodes**	**Heating Material**	**Operating Temperature Range** (**°C**)	**Reference**
				**Length** (**mm**)	**External Diameter** (**mm**)	**Internal Diameter** (**mm**)				
ZnO	Nanorod	Poly(vinyl acetate) (PVA)	Al_2_O_3_	8	2	1.6	Au	Ni–Cr	100–500	[5]
ZnO	Nanorod	Terpineol	Al_2_O_3_	–	–	–	-	-	–	[18]
SnO_2_	Nanorod	Poly(vinyl acetate) (PVA)	Al_2_O_3_	8	2	1.6	Au	Ni-Cr	100–500	[49]
SnO_2_	Nanofiber	Deionized water	–	–	–	–	Au	Ni-Cr	–	[13]
TiO_2_ (Cu-doped)	Nanofiber	Deionized water	–	–	–	–	Au	Ni-Cr	–	[50]
In_2_O_3_	Nanorod	Deionized water	Al_2_O_3_	4	1.4	1	Au	–	–	[15]
In_2_O_3_	Nanowire	Poly(vinyl acetate) (PVA)	Al_2_O_3_	8	2	1.6	Au	Ni-Cr	100–500	[51]
α-Fe_2_O_3_	Porous urchin	Terpineol	Al_2_O_3_	5	1	-	Pt	Ni–Cr	100–500	[31]

**Table 2.** Summary of various processing routes for the production of 1-D ZnO nanostructures.

**Processing Route**	**Synthesis Method**	**Starting Materials**	**Synthesis Temperature** (**°C**)	**Morphology**	**Diameter of ZnO nanostructure**	**Length of ZnO nanostructure**	**Reference**
Wet Processing route	Hydrothermal	ZnAc_2_, NaOH, absolute ethanol, distilled water	180	Nanorod	–	–	[5]
		Zn(CH_3_COO)_2_·2H_2_O, C_6_H_8_O_7_·H_2_O, absolute ethanol, distilled water	400	Nanorod (vertically aligned)	50 nm	500 nm	[7]
		Zn(NO_3_)_2_·6H_2_O, NaOH, cetyltrimethyl ammonium bromide, ethanol	120	Nanorod	–	–	[48]
		Zn(NO_3_)_2_·6H_2_O, NaOH, cyclohexylamine, ethanol, water	200	Nanorod	150–200 nm	2 μm	[52]
		Zn(SO_4_)·7H_2_O, NH_4_OH, deionized water	75–95	Nanorod	–	–	[11]
		NaOH, Zn(NO_3_)_2_, absolute ethanol, deionized water, hydroethylenediamine	180	Flowerlike	150 nm	Few micrometer	[53]
	Ultrasonic irradation in aqueous solution	Deposited Zn layer on interdigitated alumina substrate, Zn(NO_3_)_2_·6H_2_O, (CH_2_)_6_N_4_	–	Nanorod (vertically aligned)	50 nm	500 nm	[12]
Solid-state processing route	Carbothermal Reduction	ZnO powder, graphite powder, Ar gas flow, Au coated silicon substrate	900–925	Nanowire	80–120 nm	10–20 μm	[17,54]
	Solid-state chemical reaction	ZnCl_2_, NaOH, polyethylene glycol, Na_2_WO_4_·2H_2_O	RT	Nanorod	40–60 nm	200 nm	[18]
					20–40 nm	100 nm	
Vapor-7phase processing route	Thermal evaporation	Zn metal, O_2_, Ar	650–670	Nanowire	100 nm	Several microns	[55]
		Zn metal pellets, O_2_, Ar	900	Nanowire	20 nm	–	[19]
		Zn powder, O_2_, Ar	600	Nanowire	80 nm	1 μm	[56]
	Vapor-phase transport	ZnO powder, graphite, Cu catalist	930	Hierarchical dendrite	60–800 nm	–	[20]
	Aerosol	Zn powder, N_2_ gas	500–750	Fiber-mat	100–300 nm	–	[21]
				Cauliflower	20–30 nm	–	
	RF sputtering	ZnO deposited over Pt sputtered interdigitated alumina substrate	−	Nanobelt	–	Few micrometer	[22]
	Molecular beam epitaxy	Zn metal, O_3_/O_2_ plasma discharge, Au coated substrate	600	Nanorod	50–150 nm	2–10 μm	[23]

**Table 3.** Summary of the gas sensing properties of 1-D ZnO nanostructures for different gases.

**Gas Tested**	**Morphology**	**Size**		**Detection Range**	**Detection Temperature** (**°C**)	**Optimum Working Temperature** (**°C**)	**Response**			**Response Time**	**Recovery Time**	**Reference**
		**Diameter**	**Length**				**Sensitivity**	**Concentration**	**Temperature** (**°C**)			
Ethanol	Nanowire	25 ± 5	-	1–200 ppm	300	–	32 ^A^	100 ppm	300	-	-	[9]
	Nanowire	80 nm	1 μm	50–1,500 ppm	180–300	–	43 ^D^	100 ppm	300	-	-	[47]
	Nanorod (flowerlike)	150 nm	Few micron	0.5–1,000 ppm	300	–	14.6 ^A^	100 ppm	300	-	-	[53]
	Nanorod (bushlike)	15 nm	1 μm	1–1,000 ppm	300	–	29.7 ^A^	100 ppm	300	-	-	[57]
	Nanorod (vertically aligned)	50 nm	500 nm	1–100 ppm	300	–	100 ^A^	100 ppm	300	-	-	[7]
H_2_S	Nanorod	70–110 nm	0.2–1.3 μm	0.005–10 ppm	25–400	25–200	1.7 ^A^	0.05 ppm	25	-	-	[5]
	Hierarchical dendrite	60–800 nm	-	10–500 ppm	30	–	17.3 ^A^	100 ppm	30	15–20 s	30–50 s	[20]
H_2_	Nanorod (single)	-	-	1–1,000 ppm	RT	–	4% ^C^	200 ppm	RT	30–40 s	50–90 s	[11]
	Nanowire	10–30 nm	50–250 nm	100–1,000 ppm	RT	–	3 ^A^	200 ppm	RT	-	–	[59]
	Nanobelt	10 nm (thickness)	50 nm (width)	0.06–1%	150–450	385	14.3 ^C^	1%	385	48 s	336 s	[22]
	Nanorod (Pd coated)	30–150 nm	2–10 μm	10–500 ppm	RT-200	–	4.2% ^E^	500 ppm	-	-	<20 s	[23]
NO_2_	Nanowire	80–120 nm	10–20 μm	0.5–20 ppm	225	–	>95 ^B^	20 ppm	250	24 s	12 s	[54]
	Nanorods(vertically aligned)	50 nm	500 nm	10 ppb–10 ppm	150–400		824% ^F^	100 ppb	250	4.5 min	4 min	[12]
	Nanobelt	10 nm (thickness)	50 nm (width)	0.51–1.06 ppm	150–450	350	0.81 ^D^	8.5 ppm	350	180 s	268 s	[22]
	Fibre-mats	100–300	–	0.1–0.5	20–150	20	>100 ^D^	0.04	20	Order of minutes	Order of minutes	[21]
	Cauliflower	20–30				100	-		-			
Propane	Nanobelt	10 nm (thickness)	50 nm (width)	0.25–1%	150–450	370	0.17 ^C^	1%	370	72 s	252 s	[21]
HCHO (Methanal)	Nanorod	20–40 nm	100 nm	50–1,000 ppm	100–425	300	11.8 ^A^	100 ppm	300	3 s	9 s	[18]
		40–60 nm	200 nm				9 ^A^			4 s	11 s	
C_6_H_4_(CH_3_)_2_(Xylene)	Nanorod	20–40 nm	100 nm	50–1,000 ppm	100–425	150	9.6 ^A^	100 ppm	150	6 s	12 s	[18]
		40–60 nm	200 nm				6 ^A^			7 s	20 s	
CO	Nanowire	50–125 nm	1.1–5.4 μm	500 ppm	320	–	57% ^F^	500 ppm	320	–	-	[60]

Note: A: *S* = *R_a_/R_g_*, B: *S* = *R_g_/R_a_*, C: *S* = Δ*R/R_g_*, D: *S* = Δ*R/R_a_*, E: *S* = (Δ*R/R_g_*) × 100% and F: *S* = (Δ*R/R_a_*) × 100%.

**Table 4.** Summary of various processing routes for the production of 1-D SnO_2_ nanostructures.

**Processing Route**	**Synthesis Method**	**Starting Materials**	**Synthesis Temperature** (**°C**)	**Morphology**	**Diameter of SnO_2_ nanostructure**	**Length of SnO_2_ nanostructure**	**Reference**
Wet processing route	Hydrothermal	SnCl_4_.5H_2_O, NH_4_(OH), Si substrate	95	Nanowires/nanoneedle	100 nm	10–20 μm	[63]
	Hydrothermal	SnCl_4_.5H_2_O, NaOH, alcohol/water	190	Nanorod (flowerlike)	5–20 nm	100–200 nm	[64]
	Electrospinning	SnCl_2_, N,N-dimethyl formamide (DMF), ethanol, poly(vinyl pyrrolidone) (PVP)	Electrospinning: RT Calcination: 600	Nanofiber	80–160 nm	–	[13]
	Electrospinning (single capillary)	SnCl_2_.2H_2_O, ethanol, N,N-dimethylformamide, poly(vinylpyrrolidone) (PVP), PdCl_2_	Electrospinning: RT Calcination: 600	Nanofiber (Pd-doped)	200–300 nm	Tens of micrometer	[65]
		SnCl_2_.2H_2_O, ethanol, N,N-dimethylformamide, poly(vinyl pyrrolidone) (PVP), PtCl_4_	Electrospinning: RT Calcination: 600	Nanofiber (Pt-doped)	200–300 nm	–	[56]
Molten-state processing route	Molten-salt	SnO_2_ powder, NaCl, nonionic surfactant, distilled water	800	Nanorod	20–70 nm	1 μm	[16]
Solid-State Processing	Nanocarving	SnO_2_ powder, CoO powder, Au nanoparticles, H_2_, N_2_	700-800	Nanofiber	100–200 nm	–	[66]
	Direct oxidation	Sn powder, quartz tube, alumina boat, Ar, CuO, distilled water	810	Nanoribbon (with CuO nanoparticles)	20–200 nm	Order of millimeters	[27]
Vapor-phase processing route	Thermal evaporation	Sn powder, N_2_, O_2_	800	Nanowhisker	50-200 nm	Tens of micrometer	[28]
		SnO powder, Ar	1000	Nanobelt	80 nm (thickness)	330 nm (width)	[10]
		Sn powder, O_2_	800	Nanowire	40–85 nm	–	[10]
		SnO powder, Sn powder, O_2_	980, 800	Nanowire (hierarchical)	–	–	[68]
Hybrid processing route	Electrospinning, pulsed laser deposition	Zn(CH_3_COO)·2H_2_O, poly(4-vinyl phenol), ethanol, Pt interdigitated SiO_2_/Si substrate, SnO_2_	Electrospinning: 80 Calcination: 600	Nanofiber (SnO_2_ and ZnO composite)	50–80 nm	–	[67]

**Table 5.** Summary of the gas sensing properties of 1-D SnO_2_ nanostructures for different gases.

**Gas Tested**	**Morphology**	**Size**		**Detection Range**	**Detection Temperature** (**°C**)	**Optimum Working Temperature** (**°C**)	**Response**			**Response Time**	**Recovery Time**	**Reference**
		**Diameter**	**Length**				**Sensitivity**	**Concentration**	**Temperature** (**°C**)			
Ethanol	Nanowhisker	50–200 nm	Tens of micrometers	50 ppm	300	–	23 ^A^	50 ppm	300	–	10 min	[28]
	Nanorod (flowerlike)	5–20 nm	100–200 nm	10–1,000 ppm	200, 300	200	45.1 ^A^	100 ppm	200	–	–	[64]
	Nanorods (flowerlike loaded with La_2_O_3_)	5–20 nm	100–200 nm	10–1,000 ppm	200, 300	200	213 ^A^	100 ppm	200	–	–	[64]
	Nanofiber (Pd doped)	200–300 nm	Tens of micrometers	100 ppm	330–440	330	1,020.6 ^A^	100 ppm	330	<10 s	503 s for 100 ppm at 385 °C	[65]
H_2_S	Nanofiber	200–300 nm	–	4–20 ppm	300–500	300	121 ^A^	20 ppm	300	2–7 s	267–281 sfor 200 ppmat 400 °C	[56]
	Nanofiber (Pt doped)	200–300 nm	–	4–20 ppm	300–500	300	5,100 ^A^	20 ppm	300	1 s	214–267 sfor 200 ppmat 400 °C	[56]
	Nanoribbon (loaded with CuO nanoparticles)	20–200 nm	Order of millimeters	3 ppm	27–200	50	18,000 ^A^	3 ppm	50	–	–	[27]
H_2_	Nanobelts	80 nm (thickness)	330 nm (width)	2%	25–80	–	60% ^C^	2%	25	220 sat 25 °C	220 s at 25 °C	[74]
NH_3_	Nanowire	60 nm	–	300–1,000 ppm	50–300	200	11 ^A^	1,000 ppm	200	–	–	[10]
	Nanowire (hierarchical)	60 nm	–	300–1,000 ppm	50–300	200	21.7 ^A^	1,000 ppm	200	–	–	[68]
LPG	Nanowire	60 nm	–	500–2,000 ppm	50–450	350	5.8 ^A^	2,000 ppm	350	<10 s at 350 °C	<10 s at350 °C	[10]
	Nanowire (hierarchical)	60 nm	–	500–2,000 ppm	50–450	350	20.4 ^A^	2,000 ppm	350	–	–	[68]
Toluene	Nanofiber	80–160 nm	–	10–10,000 ppm	310–380	350	6 ^A^	100 ppm	350	1 s	5 s	[13]
Acetone	Nanorod	20–70 nm	1 μm	1–100 ppm	450	450	3.7 ^A^	10 ppm	450	–	–	[16]
Triethylamine	Nanorod	20–70 nm	1 μm	1–100 ppm	350	350	64.8 ^A^	50 ppm	350	<10 s	<10 s	[16]
NO_2_	Nanofiber (SnO_2_-ZnO composite)	55–80 nm	–	0.4–3.2 ppm	150–300	180–200	105 ^B^	3.2 ppm	200	–	–	[67]

Note: ^A^ *S* = *R_a_/R_g_*, ^B^ *S* = *R_g_ /R_a_*, and ^C^ *S* = (Δ*R/R_g_*) × 100%.

**Table 6.** Summary of various processing routes for the production of 1-D TiO_2_ nanostructures.

**Processing Route**	**Synthesis Method**	**Starting Materials**	**Crystal Structure**	**Synthesis Temperature (°C)**	**Morphology**	**Diameter of TiO_2_ nanostructure**	**Length of TiO_2_ nanostructure**	**Reference**
Wet processing route	Hydrothermal	TiCl_3_, HCl, NaCl, deionized water, alcohol	Rutile	200	Nanowire	20–80 nm	100–800 nm	[59]
		TiO_2_ powders, PdCl_2_, H_2_PtCl_6_, NaOH, HCl, deionized water	Lepidocrocite	150	Nanotube (with Pd/Pt nanoparticles)	100 nm	-	[75]
		TiO_2_ powders, NaOH, HCl, deionized water	Anatase	600	Nanobelt	50 nm (thickness)	100–150 nm (width)	[38]
	Hydrothermal, Photocatalytic reduction	TiO_2_ powders, NaOH, HCl, deionized water, AgNO_3_ ethanol solution	Anatase	600	Nanobelt (with Ag nanoparticles)	50 nm (thickness)	100–150 nm (width) 10–30 nm (Ag nanoparticles)	[38]
Wet processing route	Electrospinning	poly(vinyl acetate) (PVA), dimethylformamide (DMF), titanium (IV) propoxide, acetic acid	Anatase	RT	Nanofiber	120–850 nm	-	[76]
		Tetrabutyl titanate, acetic acid, ethanol, poly(vinyl pyrrolidone) (PVP), CuCl_2_.2H_2_O	Anatase, rutile, brookite	500	Nanofiber	80 nm	-	[50]
	Anodization	Titanium foil, platinum foil, hydrofluoric acid, water	Anatase, Rutile	500	Nanotube array	46–76 nm	400 nm	[44]
		Titanium foil, platinum foil, NH_4_F, (NH_4_)_2_SO_4_, deionized water.	Amorphous	450	Nanotube array	150 nm	2.3 μm	[78]
		Titanium foils, sodium hydrogen sulfate monohydrate, potassium fluoride, sodium citrate tribasic dehydrate, sodium hydroxide	-	370-630	Nanotube array	30–110 nm	380 nm −6 μm	[79]
		Titanium foil, acetone, isopropanol, platinum foil, NH_4_F, dimethyl sulphoxide	Anatase	400	Nanotube array	350 nm	3.5 μm	[14]
	Anodization, hydrothermal	Titanium foil, acetone, isopropanol, platinum foil, NH_4_F, dimethyl sulphoxide, HCl, titanium (IV) isopropoxide, ethanol	Anatase	400	Nanotube (branched array)	350 nm	3.5 μm	[14]
		Titanium foil, acetone, isopropanol, platinum foil, NH_4_F, dimethyl sulphoxide, P25, distilled water	Anatase	400	Nanotube array (P25 coated)	350 nm	3.5 μm	[14]
Solid-state etching	Nanocraving	TiO2 powder, H_2_, N_2_	Rutile	700	Nanofiber	15–50 nm	1–5 μm	[80]
		TiO_2_ powder, mixed oxide of TiO_2_ and SnO_2_ powder, isopropanol, H_2_, N_2_	Rutile	700	Nanofiber	5–10 nm	0.2–1 μm	[25]
Solid-state etching	UV lithography, dry plasma etching	TiO_2_, positive photoresist, silicon substrate	Anatase	500	Nanowire	90-–180 nm	1400 μm	[26]

**Table 7.** Summary of the gas sensing properties of 1-D TiO_2_ nanostructures.

**Gas Tested**	**Morphology**	**Crystal Structure**	**Size**		**Detection Range**	**Detection Temperature** (**°C**)	**Optimum Working Temperature** (**°C**)	**Response**			**Response Time**	**Recovery Time**	**References**
			**Diameter**	**Length**				**Sensitivity**	**Concentration**	**Temperature** (**°C**)			
H_2_	Nanotube array	Anatase, rutile	46–76 nm	400 nm	100 ppm-4%	180–400	–	∼1,000 ^A^	1,000 ppm	400	3 min	–	[44]
	Nanotube array	–	30–110 nm	380 nm–6 μm	1,000 ppm	–	–	∼10^9 A^	1,000 ppm	–	–	–	[79]
	Nanowire	Rutile	20–80 nm	100–800 nm	100–1,000 ppm	RT	–	8 ^A^	1,000 ppm	RT	–	–	[59]
	Nanotube (loaded with Pt and Pd nanoparticles)	Lepidocrocite	100 nm	–	0.5–3%	25–350	250	–	–	–	–	–	[75]
	Nanofiber	Rutile	5–10 nm	0.2–1 μm	0.5–2%	300–600	–	1.25 ^B^	2%	400	1–2 min	5–7 min	[25]
Ethanol	Nanowire array	Anatase	90–180 nm (Width)	1400 μm	0.3–3%	500–600	–	50 ^A^	2%	550	–	–	[26]
	Nanobelt	Anatase	50 nm (thickness)	100–150 nm (width)	20–500 ppm	150–400	200–250	46.153 ^A^	500 ppm	200	1–2 s	1–2 s	[38]
CO	Nanofiber	Anatase, rutile, brookite	80 nm		5–1,600 ppm	260–340	300	21 ^A^	100 ppm	300	4 s	8 s	[50]
NO_2_	Nanofiber	Anatase	120–850 nm	–	50–250 ppb	300, 400	300	74.3 ^A^	250 ppb	300	0.8 min at 300 °C for 250 ppb	4.4 min at 300 °C for 250 ppb	[76]
O_2_	Nanotube array	Amorphous	150 nm	2.3 μm	200 ppm-20%	50–300	100	∼100 ^C^	–	100	–	–	[78]

Note: ^A^ *S* = *R_a_/R_g_*, ^B^ *S* = *R_g_ /R_a_*, and ^C^ *S* = Δ*R/R_a_*.

**Table 8.** Summary of various processing routes for the production of 1-D In_2_O_3_ nanostructures.

**Processing Route**	**Synthesis Method**	**Starting Materials**	**Synthesis Temperature** (**°C**)	**Morphology**	**Diameter of TiO_2_ nanostructure**	**Length of TiO_2_ nanostructure**	**Crystal Structure**	**Reference**
Wet processing route	Electrospinning	In(NO_3_)_3_.4.5H_2_O, N, N-dimethylformamide, ethanol, poly(vinyl pyrrolidone) (PVP)	Nanowire: RT Calcination: 500-800 °C	Nanofiber	60–100 nm	Tens of micrometers	Cubic	[92]
		Nanofiber growth: In(NO_3_)_3_.4.5H_2_O, N, N-dimethylformamide, ethanol, poly(vinyl pyrrolidone) (PVP) Pt deposition: H_2_PtCl_6_, sodium citrate, deionized water	Nanowire: RT Calcination: 700 °C	Nanofiber (loaded with Pt (nanoparticles)	60–100 nm	Tens of micrometers	Cubic	[93]
	Sol-gel	InCl_3_.4H_2_O, sodium dodecyl sulfate, NaOH, deionized water	60 °C	Nanorod	70–100 nm	300–900 nm	Cubic	[15]
Solid-state processing route	Carbothermal reduction	In_2_O_3_ powder, active carbon, alumina boat, N_2_	1000 °C	Nanowire	60–160 nm	0.5 to few micrometer	-	[94]
Vapor-phase processing route	CVD	Indium grains, alumina boat, quartz tube, silicon wafer coated with 10 nm Au layer, Ar gas	800	Nanowire	70–80 nm	Several micrometer	Cubic	[29]
			900	Nanoneedle	150–200 nm	4–5 μm		
		Indium particles, alumina boat, quartz tube, silicon wafer, Ar, O_2_	800	Nanopushpin	80–120 nm	500 nm–1μm	Cubic	[30]
Vapor-phase processing route	CVD	Nanofiber growth: In_2_O_3_ with graphite powder, Ar gas, Silicon wafer with Au layer Au deposition: HAuCl_4_.3H_2_O, sodium citrate, hydrogen peroxide, NH_4_OH, p-aminophenyltrimethoxysilane, toluene, N_2_, acetone, deionized water	Nanofiber growth: 900 °C Au deposition: 115 °C	Nanofibers (loaded with Au nanoparticles)	150–200 nm	–	–	[95]
	CVD and sputtering	Nanowire growth: In powders, Mg nanopowders, silicon substrate with Au layer, quartz tube, Ar gas, O_2_ gas. Pt deposition: Turbo sputter coater, Pt target, Ar gas	Nanowire: 800 °C Pt deposition: RT Annealing: 800 °C	Nanowire	–	–	Cubic	[24]
Hybrid processing route	Solvothermal	Oleic acid, n-amyl alcohol, n-hexane, In(NO_3_)_3_, NaOH, absolute ethanol and distilled water.	InOOH: 200 °C In_2_O_3_: 600 °C	Nanorod	20–50 nm	>100 nm	-	[94]

**Table 9.** Summary of the gas sensing properties of 1-D In_2_O_3_ nanostructures.

**Gas Tested**	**Morphology**	**Crystal Structure**	**Size**		**Detection Range**	**Detection Temperature** (**°C**)	**Optimum Working Temperature** (**°**)	**Response**			**Response Time**	**Recovery Time**	**Reference**
			**Diameter**	**Length**				**Sensitivity**	**Concentration**	**Temperature** (**°**)			
H_2_	Nanorod	Cubic	70–100 nm	300–900 nm	50 × 10^–6^–5,000 × 10^–6^	250–450	340	∼6 ^A^	500 × 10^–6^	340	6 s	6 s	[15]
	Nanowire	Cubic	70–80 nm	Several micrometer	500–1,500 ppm	150–400	–	–	–	–	31 s	80 s	[29]
	Nanoneedle	Cubic	150–200 nm	4–5 μm	500–1,500 ppm	150–400	–	–	–	–	60 s	–	[29]
	Nanopushpin	Cubic	80–120 nm	500 nm–1 μm	500–1,500 ppm	150–400	–	–	–	–	35 s	60 s	[30]
H_2_S	Nanofiber	Cubic	60–100 nm	–	50–600 ppm	140–300	260	150 ^A^	600 ppm	260	–	–	[93]
	Nanofiber (loaded with Pt nanoparticles)	Cubic	60–100 nm	–	50–600 ppm	140–300	200	1,490 ^A^	600 ppm	200	60 s	120 s	[93]
C_2_H_5_OH	Nanofiber	Cubic	60 nm	–	100–15,000 ppm	260–340	300	379 ^A^	15,000 ppm	300	1 s	5 s	[92]
	Nanowire	–	60–160 nm	0.5 to a few micrometer	100–1,000 ppm	150–400	370	25.3 ^A^	1,000 ppm	370	10 s	20 s	[94]
	Nanorod	–	20–50 nm	>100 nm	50–1,000 ppm	330	–	11.5 ^A^	50 ppm	330	6 s	11 s	[94]
CO	Nanowire (functionalized with Au nanoparticles)		150–200 nm	–	0.2–5 ppm	RT	–	∼104 ^A^	5 ppm	RT	130 s	50 s	[95]
O_2_	Nanowire	Cubic	–	–	10–400 ppm	50	–	–	–	–	100 s	–	[24]

Note: ^A^ *S* = *R_a_/R_g_*.

**Table 10.** Summary of various processing routes for the production of 1-D nanostructures of non-convention sensors.

**Materials**	**Synthesis Method**	**Starting Materials**	**Crystal Structure**	**Synthesis Temperature** (**°C**)	**Morphology**	**Diameter of TiO_2_ Nanostructure**	**Length of TiO_2_ nanostructure**	**Reference**
WO_2.72_	Solvothermal	Tungsten hexachloride, ethanol	Monoclinic	200	Nanowire	5–30 nm	100–500 nm	[59,96]
WO_3_	Thermal oxidation	SiO_2_/Si substrate, porous single wall carbon nanotubes, arc-discharge chamber, DC sputtering, tungsten target, tube furnace	Monoclinic	700	Nanowire	70 nm	Few micrometer	[97]
β-AgVO_3_	Hydrothermal	V_2_O_5_ powder, Ag_2_O powder, distilled water	Monoclinic	180	Nanowires	50–100 nm (thickness)	100–700 nm (width)	[34,35]
CdO	Hydrothermal	CdCl_2_·2.5H_2_O, ethylenediamine, Na_2_CO_3_, NH_3_, distilled water, ethanol	Cubic	Nanowire: 180 Calcination: 300–650	Nanowire	120 nm	100μm	[99]
MoO_3_	Sol-gel	Molybdenum iso-propoxide	–	–	Nanoneedle	–	–	[39]
MoO_3_	Thermal evaporation	MoO_3_ powder, O_2_, Ar	Orthorhombic	770	Lamellar	500 nm (thickness)	5 μm (width)	[32]
CuO	Hydrothermal	Sodium dodecylbenzenesulfonate, CuSO_4_, NaOH, distilled water, absolute ethanol, H_2_PtCl_6_, HAuCl_4_, L-ascorbic acid, absolute ethanol, distilled water	–	120	Nanoribbons (loaded with Pt and Au)	2–8 nm (thickness)	30–100 nm (width)	[101]
TeO_2_	Thermal evaporation	Te metal, alumina crucible, silicon wafer	Tetragonal	400	Nanowire	30–200 nm	Tens of micrometers	[102]
α-Fe_2_O_3_	Hydrothermal	FeSO_4_.7H_2_O, CH_3_COONa.4H_2_O, deionized water, absolute alcohol	Hexagonal	500	Porous urchin	30–40 nm	500 nm	[31]

**Table 11.** Summary of the gas sensing properties of various 1-D nanostructures for non-conventional sensing oxides.

**Materials and Morphology**	**Gas Tested**	**Crystal Structure**	**Size**		**DetectionRange**	**Detection Temperature** (**°C**)	**Optimum Working Temperature** (**°C**)	**Response**			**Response Time**	**Recovery Time**	**Reference**
			**Diameter**	**Length**				**Sensitivity**	**Concentration**	**Temperature** (**°C**)			
WO_2.72_ Nanowire	H_2_	Monoclinic	5–30 nm	100–500 nm	100–1,000 ppm	25	–	22 ^A^	1,000 ppm	25	38 s	26 s	[59]
WO_2.72_ Nanowire	LPG	Monoclinic	5–30 nm	100–500 nm	100–1,000 ppm	25	–	15 ^A^	1,000 ppm	25	38 s	26 s	[59]
WO3 Nnowire	NH_3_	Monoclinic	70 nm	Few micrometer	300–1,500 ppm	200–300	250	9.67 ^A^	1,500 ppm	250	7 s	8 s	[97]
β-AgVO_3_ Nanowire	H_2_S	Monoclinic	50–100 nm (thickness)	100–700 nm (width)	50–400 ppm	250	–	>1.12 ^A^	400 ppm	250	<10 s	<20 s	[35]
CdO Nanowire (porous)	NO_x_	Cubic	120 nm	100 μm	1–300 ppm	100	–	>150 ^C^	150 ppm	100	–	–	[99]
MoO_3_ Needle	O_2_	–	–	–	1,000 ppm	370	–	39 ^C^	1,000 ppm	370	1 s	5 s	[39]
MoO_3_ Lameller	NO_2_	Orthorhombic	500 nm (thickness)	5 μm (width)	0.6–10 ppm	180–300	225	1.18 ^C^	10 ppm	250	–	–	[100]
CuO Nanoribbon	HCHO	–	2–8 nm	30–100 nm	5–500 ppm	200	–	∼4 ^B^	500 ppm	200	2–4 s	3–7 s	[101]
CuO Nanoribbon (Au loaded)	HCHO	–	2–8 nm	30–100 nm	5–500 ppm	200	–	∼5.5 ^B^	500 ppm	200	–	–	[101]
CuO Nanoribbon (Pt loaded)	HCHO	–	2–8 nm	30–100 nm	5–500 ppm	200	–	∼8 ^B^	500 ppm	200	–	–	[101]
CuO Nanoribbon	Ethanol	–	2–8 nm	30–100 nm	5–1,000 ppm	200	–	∼3.5 ^B^	1,000 ppm	200	3–6 s	4–9 s	[101]
CuO Nanoribbon (Au loaded)	Ethanol	–	2–8 nm	30–100 nm	5–1,000 ppm	200	–	∼3.5 ^B^	1,000 ppm	200	–	–	[101]
CuO Nanoribbon (Pt loaded)	Ethanol	–	2–8 nm	30–100 nm	5–1,000 ppm	200	–	∼6 ^B^	1,000 ppm	200	–	–	[101]
TeO_2_ Nanowire	NO_2_	Tetragonal	30–200 nm	Tens of micrometer	10–100 ppm	26	–	–	–	–	2 min	–	[32]
α-Fe_2_O_3_ Porous urchin	H_2_S	Hexagonal	30–40 nm	500 nm	1–100 ppm	150–450	250 (n-type response)	∼2.5 ^C^	100 ppm	250	5 s	10 s	[31]

Note: ^A^ *S* = *R_a_/R_g_*, ^B^ *S* = *R_g_ /R_a_* and ^C^ *S* = Δ*R/R_a_*.

**Table 12.** Summary of the gas sensing properties of 1-D nanostructures for various gases.

**Gas Tested**	**1-D Nanostructure**	**Optimum operating Temperature (°C)**	**Sensitivity**	**Gas concentration**	**Reference**
Ethanol	ZnO Nanorod (vertically aligned)	300	100	100 ppm	[7]
	SnO2 Nanorod (flowerlike)	200	45.1	100 ppm	[64]
	SnO2 Nanorod (flowerlike loaded with La2O3)	200	213	100 ppm	[64]
	SnO2 Nanofiber (Pd doped)	330	1,020.6	100 ppm	[65]
H2S	ZnO Hierarchical dendrite	30	17.3	100 ppm	[20]
	SnO2 Nanofiber	300	121	20 ppm	[56]
	SnO2 Nanofiber (Pt doped)	300	5,100	20 ppm	[56]
	SnO2 Nanoribbon (loaded with CuO nanoparticles)	50	18,000	3 ppm	[27]
	In2O3 Nanofiber (loaded with Pt nanoparticles)	200	1,490	600 ppm	[93]
H2	TiO2 Nanotube array	-	109	1,000 ppm	[79]
CO	TiO2 Nanofiber	300	21	100 ppm	[50]
	In2O3 Nanowire (functionalized with Au nanoparticles)	RT	104	5 ppm	[95]

Note: Sensitivity; *S* = *R_a_/R_g_*.
